# Perceiving and Countering Marine Biofouling: Structure, Forces, and Processes at Surfaces in Sea Water Across the Length Scales

**DOI:** 10.1021/acs.langmuir.5c00450

**Published:** 2025-03-20

**Authors:** Xiaoyan Xu, Shifeng Guo, Gyula Julius Vancso

**Affiliations:** † Shenzhen Key Laboratory of Smart Sensing and Intelligent Systems, Shenzhen Institute of Advanced Technology, Chinese Academy of Sciences, Shenzhen 518055, P.R. China; ‡ University of Chinese Academy of Sciences, Beijing 100049, China; § Guangdong Provincial Key Lab of Robotics and Intelligent System, Shenzhen Institute of Advanced Technology, Chinese Academy of Sciences, Shenzhen 518055, P.R. China; ∥ The Key Laboratory of Biomedical Imaging Science and System, Chinese Academy of Sciences, Shenzhen 518055, P.R. China; ⊥ School of Materials Science and Engineering, Nanyang Technological University, Singapore 639798, Singapore; # Sustainable Polymer Chemistry & Materials Science and Technology of Polymers, MESA+, Institute of Nanotechnology, University of Twente, P.O. Box 217, 7500 AE Enschede, The Netherlands

## Abstract

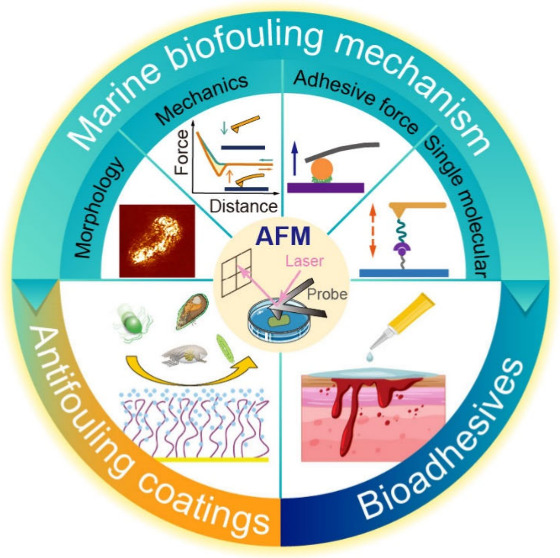

In marine industries, severe economic losses are caused by accumulating organisms on surfaces in biofouling processes. Establishing a universal and nontoxic protocol to eliminate biofouling has been a notoriously difficult task due to the complexity of the marine organisms’ interactions with surfaces and the chemical, mechanical, and morphological diversity of the surfaces involved. The tremendous variety of environmental parameters in marine environments further complicates this field. For efficient surface engineering to combat fouling, secretion, chemical structure, and properties of biobased adhesives and adhesion mechanisms must be understood. Advanced characterization techniques, like Atomic Force Microscopy (AFM), now allow one to study the three parameters determining surface adhesion and, eventually, fouling, i.e., morphology, chemistry, and surface mechanical modulus. By AFM, characterization can now be performed across length scales from nanometers to hundreds of micrometers. This review provides an up-to-date account of the most promising AFM-based approaches for imaging and characterizing natural adhesives provided by marine organisms. We summarize the current understanding of the molecular basis and the related relevant processes of marine fouling. We focus on applications of AFM “beyond imaging”, i.e., to study interactions between adhesives and the surfaces involved. Additionally, we discuss the performance enhancement of polymer antifouling coatings using information derived from AFM. Knowledge and control of marine adhesion can be applied to prevent marine fouling, as well as to design bioadhesives to enhance potential medical applications. We present some milestone results and conclude with an outlook discussing novel possibilities for designing antifouling coatings and medical bioadhesives.

## Introduction

**Figure 1:**
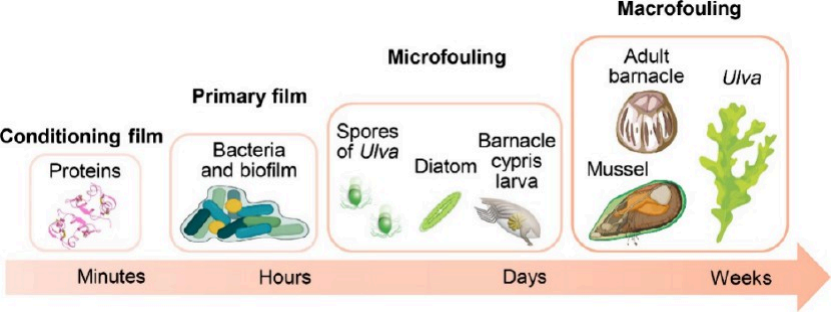
Schematic illustration of the typical marine fouling organisms and marine biofouling process timeline. Schematics not drawn to scale.

Marine fouling refers to the undesirable attachment, growth, and accumulation of marine organisms on submerged surfaces in marine environments, including ship hulls, jetties, and other marine facilities.^1−4^ In general, marine fouling occurs during steps in succession, in which surface-adsorbed organic molecules attract bacteria, creating a biofilm. This is followed in time by the subsequent settlement of micro and macro-foulers within several weeks (Figure 1).^5,6^ However, some organisms, like barnacle cypris larvae and spores of seaweeds, are capable of settling on pristine surfaces without biofilm.^7,8^ Marine fouling inevitably occurs on the surface of almost any material immersed in seawater.^9−12^

To prevent marine fouling, several strategies have been devised, including the use of (toxic) agents, surface engineering (patterning), and surface modulus engineering for fouling release. Currently, antifouling coatings generally employ three major strategies, i.e., the use of biocides, the engineering of antifouling surfaces, and the application of fouling release coatings. These aim at preventing the attachment of marine organisms, or weakening the adhesion of fouling organisms for easy removal.^13^ Biocides, including coatings such as tributyltin (TBT) have been efficient and broad-spectrum antifouling methods. However, TBTs were banned in 2008 due to their deleterious effects on nontarget organisms.^14^ Since then, alternatives containing other metal compounds (e.g., copper-based and zinc-based) have been applied, facing increasingly rigorous scrutiny due to potential threats to marine ecosystems.^15^ Inspired by the resistance of natural organisms to fouling, a series of microtopographical surfaces with different shapes and sizes have been fabricated for marine antifouling, including the textured surface, hierarchical structure, dynamic surface, and PEGylated coatings.^16−18^ Although these approaches promise eco-friendly antifouling, they face some practical restrictions such as high cost, short lifetime, and narrow biological spectrum of efficiency. The currently employed antifouling approaches have been well summarized, along with their strengths and limitations.^13,19−21^

There are over 4000 fouling species in the oceans.^22^ However, the marine environmental conditions in different regions are complex and varied in terms of salinity, temperature, pH, flow rates, biosphere, etc., which also makes the dominant fouling organisms different.^22,23^ Therefore, designing a universal coating has been a tremendous challenge (and likely impossible), as different fouling organisms rely on different adhesion mechanisms, have different affinities to various surfaces, and respond differently to the antifouling strategies chosen. Yet, at least different types of foulants can be arranged in groups, and group-specific prevention could be achieved. For example, Flammang et al. found that the composition of adhesives secreted by various marine fouling organisms is complex but predominantly made up of proteins.^24^ In mussels, for instance, adhesion proteins, including catechols (dopamine segments), have been validated as key substances for strong surface adhesion.^25^ These studies suggest that in such cases, inhibiting the adsorption of adhesives can prevent biofouling attachment, as adhesives mediate the adhesion behavior of the fouling organisms. However, it should be remembered that this applies to only a specific group of foulants.

As mentioned, three basic parameters govern the fouling attachment: surface morphology, mechanical properties, and chemical composition. Exploration of the nanomechanical properties of the adhesive and its interaction with the surface, of course, would be one of the pivotal points for understanding the first steps of the fouling process and facilitating the design of more effective antifouling coatings. Surface morphology patterning and control of chemical composition are the two other key parameters, and all these could be efficiently studied across the length scales by different types of imaging using Atomic Force Microscopy (AFM) by adjusting the surface chemical and physicochemical properties. As mentioned, understanding, design, and synthesis of biobased proteinaceous adhesives also hold great promise in bioadhesives, e.g., for surgical applications, medical implants, and bioelectronics.

**Figure 2:**
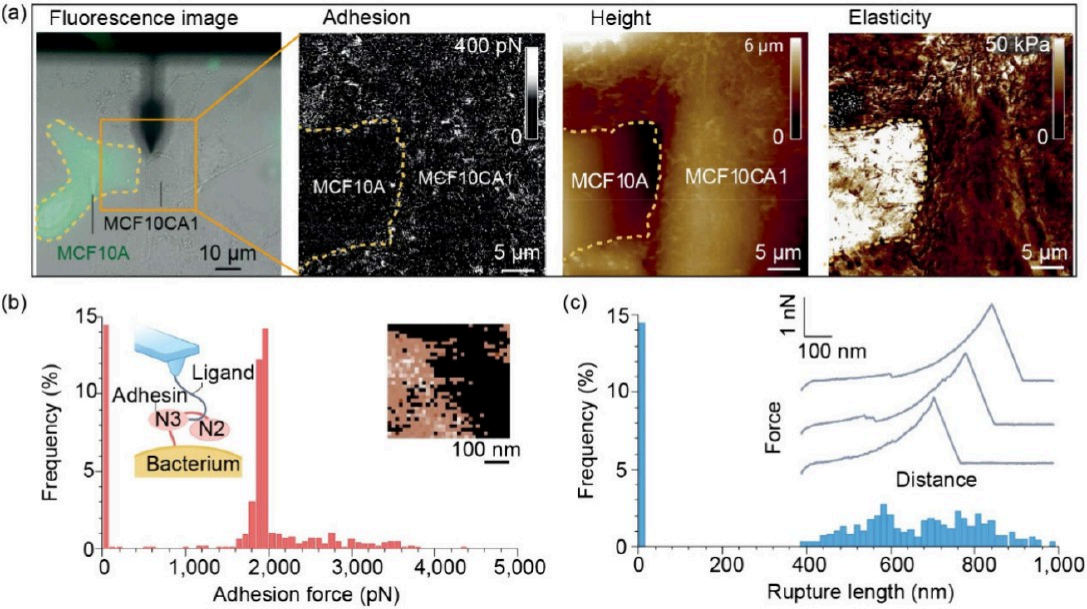
(a) Fluorescence microscopy image of adjacent MCF10A (healthy) and MCF10CA1 (malignant) cells and correlated adhesion, height, and elasticity maps. Reproduced from ref (41). Available under a Creative Commons CC-BY License. Copyright 2020 The Authors. Published by Wiley-VCH GmbH. (b) The adhesion force histogram plot of single-molecule force spectroscopy data as recorded on a living bacterium, inset: each colored pixel represents an adhesin-ligand binding event.^35^ (c) A rupture length histogram plot of single-molecule force spectroscopy data in (b), inset: the representative force curves. Reproduced with permission from ref (35). Copyright 2021 Springer Nature.

Over the past three decades, AFM has proven to be a versatile tool for qualitative and quantitative characterization of biomolecular/interface interactions.^26,27^ AFM-based force spectroscopy allows mechanical, chemical, electrostatic, and biological properties of surfaces to be efficiently explored with high accuracy.^28^ As exemplified in Figure 2(a), the adhesive, height, and elastic properties of both healthy and malignant cells can be distinctly characterized by AFM. When probing materials, the AFM tip probe is in the close vicinity of the sample or even in contact with it. The contact can be stable (in contact modes) or intermittent (in tapping mode operations). AFM cantilever deflection measurements quantify interaction forces between the AFM tip probe and surfaces. Data obtained is converted using various calibration approaches into force–distance (FD) curves, such as AFM-based force spectroscopy.^28,29^ Force distance data can be used to characterize surface mechanical properties (e.g., by quantitative nanomechanics).^30^ Various force spectroscopy approaches have also been pivotal to understanding molecular interactions between various targets, including specific functional groups to small organic molecules, macromolecules (such as proteins, DNA, polysaccharides, enzymes, etc.),^31,32^ bacteria,^33,34^ cells,^35^ and other colloidal sized targets. As a common method for studying interactions, one of the targets is attached to the AFM probe, while the other is located on the substrate. For single-molecule force spectroscopy, there is ideally only one pair of molecules in effective contact between the tip and the sample, which can be achieved by adjusting the target surface molecular density. If macromolecules are studied, statistical analyses are employed to derive forces at the single molecule level (stretching or rupture and forming of bonds).^36^Figure 2(b) illustrates the adhesion force histogram plot of single-molecule force spectroscopy data recorded on a living bacterium, with ∼2 nN force characteristic of single molecular interaction. In Figure 2(c), the corresponding rupture length histogram plot further elucidates the data from Figure 2(b), with its inset presenting representative force curves, further demonstrating the power of AFM in characterizing molecular interactions with high precision.

In this contribution, we summarize only the most promising AFM-based techniques that can be used to better understand the interactions between marine fouling organisms and surfaces in a subsequent section. We begin by introducing major marine fouling organisms and their associated fouling mechanisms, then continue exploring how AFM aids in studying the morphology and mechanical properties of natural adhesives. Starting with mesoscale studies using colloidal probes, which offer insights into protein–surface adhesion forces at the mesoscale, we delve into molecular-scale interactions, including single-molecule force spectroscopy and modifications of AFM probes. Subsequently, we present applications of AFM in characterizing antifouling coatings and bioadhesives. Finally, artificial intelligence-assisted AFM data analysis and material design are summarized. Each section starts with a discussion of relevant theoretical concepts before transitioning to examples of practical applications. We note that there is a very large number of review papers and even books, have been published in the literature, providing the physical foundations needed to tackle the applications of our focus.^27,29,37−40^

## Major Marine Fouling Organisms and Related Fouling Mechanisms

Marine fouling organisms have evolved sophisticated adhesion strategies to thrive in the dynamic marine environment, particularly in tidal and intertidal zones. Biofilms consist of adsorbed molecules such as proteins, attached remnants of marine species, bacteria, diverse microorganisms, and their extracellular polymeric constituents (EPS).^42,43^ The EPS matrix, formed by a combination of proteins, polysaccharides, nucleic acids, and lipids, provides structural integrity and a protective environment for the biofilm community.^44^ To this layer complex, heterogeneous communities of fouling species become attached in the next stage of the process. These communities consist of a diverse array of microorganisms, diatoms, spores, and microscopic foulants and form the microfouling stage in the process. Finally, larger soft and hard species develop to form the macrofouled surface (see Figure 1). The bacteria within the biofilm are often the initial colonizers, utilizing flagella or secreted EPS to bridge the gap between the bacteria and the surface, leading to irreversible adhesion.^45^ Diatoms, a significant group of unicellular microalgae within biofilms, are notable for their robust adhesion to submerged surfaces, including antifouling coatings. They play a crucial role in the adhesion process through the secretion of EPS, which typically exhibit higher concentrations of sulfates and glucose than those produced by bacteria.^46,47^ For raphid diatoms, their movement and adhesion are facilitated by secreting EPS through specialized structures like the raphe.^48^ Apart from diatoms, some species of green seaweeds (e.g., *Ulva*) are prevalent algal biofoulers in the ocean. *Ulva* spores are pear-shaped, 7–8 μm in size, forming a bulge called the apical papilla between the four flagella (see Figure 1).^48^ Swimming spores utilize their apical papilla for surface contact during localized searching. At this stage, they may rotate and temporarily attach to the surface by releasing a thin layer of elastic material.^49^ Once a suitable surface is identified, the spores permanently anchor themselves by deploying glycoprotein adhesives, and the settled spores germinate into new adult plants within 24 h.^50^ This process, from settlement to adhesion, is rapid, lasting only seconds to minutes, ensuring the spores’ capacity for swift and robust attachment to a favorable surface.

**Figure 3:**
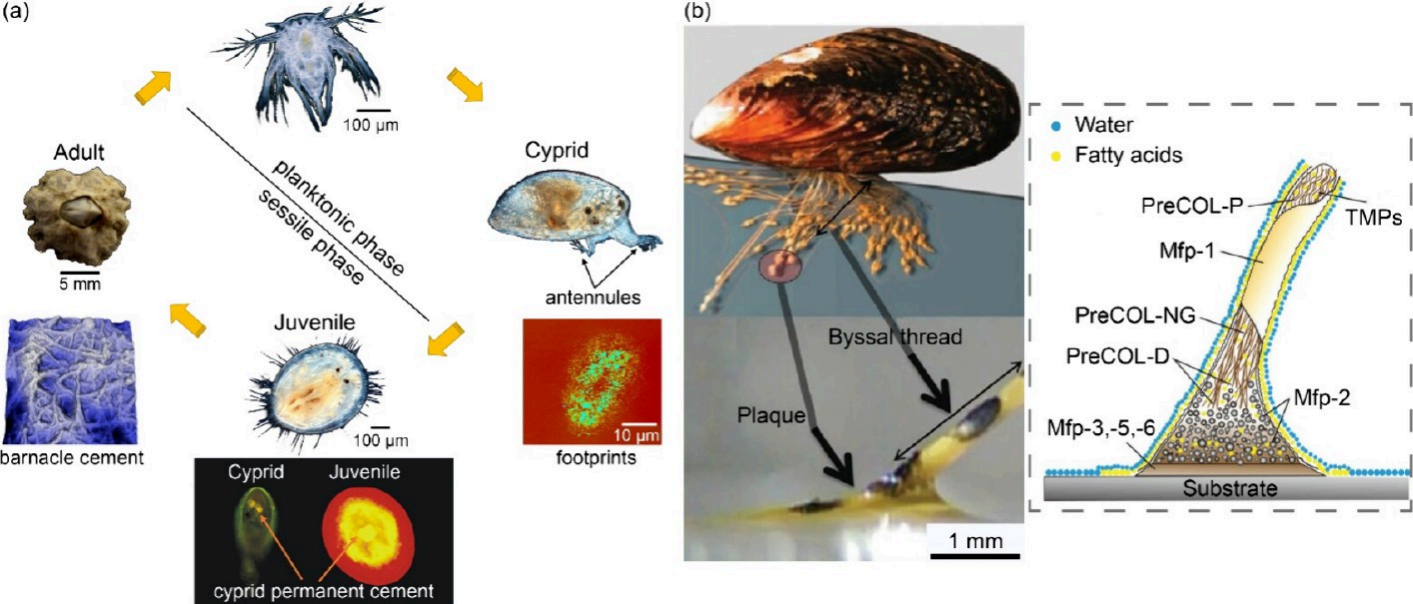
Two marine organisms secreting underwater adhesive. (a) The life cycle of a barnacle and the adhesives secreted at different stages. (b) schematic of the mussel byssal thread adhesive mechanism. Reproduced with permission from refs (59 and 60). Copyright 2018 and 2021 Royal Society of Chemistry.

Barnacles, notorious as hard macrofoulers, have been widely investigated.^4,51^ Following six nauplius stages in the planktonic phase, the cyprids are capable of temporary attachment and site exploration for finding ultimate settlement positions (Figure 3(a)). During surface exploration, the discs on the third segment of the antennules secrete temporary proteinaceous adhesives known as “footprints,” enabling cyprids to reversibly adhere to hard substrates.^52^ Once a suitable site for adult development has been identified, the cyprid secretes permanent adhesives and undergoes metamorphosis into an adult barnacle.^53^ Another hard fouler, mussels that belong to the class of bivalve mollusks, can adhere to a rigid substrate by producing a bundle of elastic byssus threads (Figure 3(b)).^54^ Each byssus thread (typically ranging from 2 to 4 cm in length and 0.10 to 0.15 mm in diameter)^55^ is produced from the ventral groove of the foot. It forms an expanded plaque at the distal end, which mediates adhesion to the substrate interface.^24^ Before attaching a new thread, the mussel’s foot protrudes from the shell and explores the surfaces with a radius of approximately 5–6 cm.^56^ Upon finding a suitable area, the mucous material is transferred to the substrate for adhesion, yet mussels retain the capability to change anchoring positions by detaching their foot and generating new byssus threads.^54^ The mussel adhesive composition comprises special proteins and is well-characterized. The most essential units for adsorption feature catechols, with 3, 4-dihydroxyphenylalanine (DOPA) being a common feature in the byssal plaques.^57,58^ DOPA not only mediates physicochemical interactions with the surface but also acts as a cross-linking agent, enhancing the cohesive strength of the foot proteins.^56^ As shown in Figure 3(b), mussel adhesion is a dynamic process with time-regulated secretion,^25^ and the foot first releases fatty acids at the interface to repel water from the surface in preparation for subsequent adhesive secretion and adhesion.^59^

## The Use of AFM to Study the Morphology of the Natural Adhesives of Fouling Organisms

We first focus our attention on the morphology of natural adhesives and the possibility of obtaining unique information about them through AFM. Here we refer to the morphology of an adhesive to its physical structure, which can include the shape, size, and arrangement of the adhesive components at the micro or nanoscale. Understanding the morphology of natural adhesives is essential as it plays a vital role in anchorage for an organism’s entire lifespan. When studying this morphology, the characterization method mustn’t alter or damage the intrinsic features of the morphology. AFM with “gentle” imaging modes (e.g., tapping) offers such possibilities and allows one to directly visualize the morphology of the adhesives across the length scales, providing insights into the design principles of nature’s adhesion strategies. Natural adhesives secreted by the fouling organisms can exhibit a variety of structures, such as nanopillars, nano- or microfibers, or gel-like matrices. AFM is a reliable tool for high-resolution imaging of soft biomaterials under physiological conditions without sample fixation, dehydration, or staining. Below, we discuss some representative examples to illustrate the employment of AFM in visualizing morphology.

**Figure 4:**
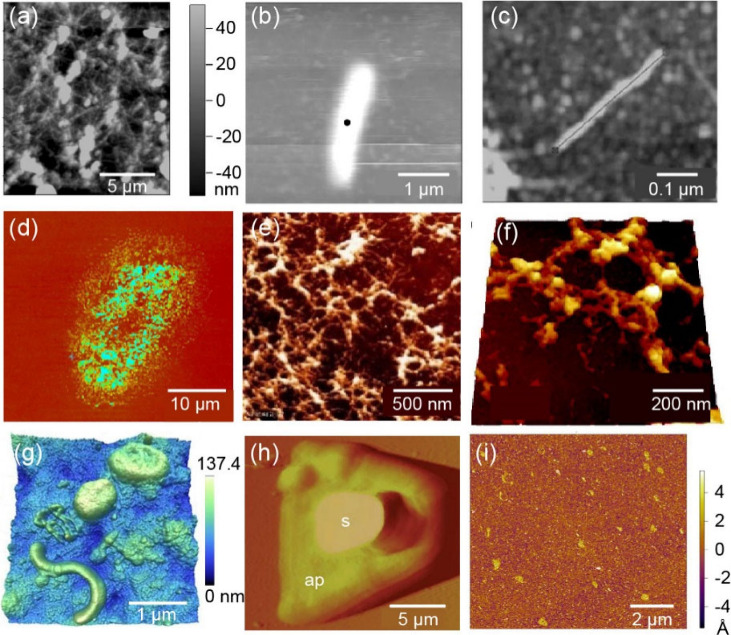
AFM morphological images of the natural adhesives. (a) Mesh morphology of the barnacle cement in seawater. (b) A larger, more regular rod-shaped structure comprising the mesh. (c) Smaller rod-like and smaller globular features. (a–c) Adapted with permission from ref (63). Copyright 2009 Taylor and Francis Ltd. (d) Morphology of cyprid footprints deposited on a hydrophilic surface. Reprinted from ref (65). Copyright 2014 American Chemical Society. (e) The deposited footprint material is fibrillar in appearance, and nanofibrils vary in height between 7 and 150 nm. Reprinted with permission from ref (67). Copyright 2008 Royal Society. (f) Cyprid footprint deposited on -NH_2_ surface. A micrograph imaged in the air indicates that the footprint protein exhibits an aggregated fibrillar structure. Reprinted with permission from ref (68). Copyright 2009 Taylor and Francis Ltd. (g) AFM images of the natural marine bacterial community. Adapted with permission from ref (70). Copyright 2010 Oxford University Press. (h) A settled zoospore showing the adhesive pad (ap) surrounding the original spore (s). Reprinted with permission from ref (71). Copyright 2000 Springer Nature. (i) AFM images of mussel adhesive proteins (Pvfp-5) on mica. Reprinted from ref (25). Available under a Creative Commons CC-BY License. Copyright 2015 The Authors. Published by Springer Nature.

Dufrêne et al. demonstrated the utility of AFM in directly visualizing the surface ultrastructure of living microbial cells, revealing distinct morphological features such as the uniform rodlets coverage on dormant spores and the smooth surface on germinating spores.^61^ Rittschof et al. focused on imaging the topography of barnacle cement, discovering closely interlocking fibers with a diameter range of 2 to 25 nm on the barnacle baseplate in air.^62^ As shown in Figure 4(a), the mesh appearance when the cement is scanned over a large area in seawater was reported by Walker et al.^63^ A rod-like structure can be observed by further enlarging the mesh structure (Figure 4(b)). Figure(c) presents a diminutive rod-like feature with a diameter of 11 nm and a length of 300 nm. Berglin and Gatenholm revealed that different substrate properties strongly affect the morphology of the barnacle adhesive plaque.^64^ They found that on the polydimethylsiloxane (PDMS) substrate with low modulus and surface energy, the synthesized adhesive plaque of barnacle exhibited a completely granular morphology, whereas a continuous film-like appearance with a few fused granules was observed on poly(methyl methacrylate) (PMMA) with high modulus.

As mentioned above, temporary adhesion is fundamental to the settlement of cyprids and their subsequent metamorphosis into adult barnacles. Understanding the temporary adhesion mechanism is, therefore, crucial for effectively preventing barnacle adhesion. Researchers have suggested that footprints facilitate temporary adhesion and serve as a settlement cue in cyprids.^5^ However, due to the small distribution and optical transparency of footprint proteins, obtaining their morphological information has been challenging for a long time. AFM can image the surface morphology of soft materials that span nanometers to hundreds of microns, making it an ideal tool for studying cyprid footprints.

Guo et al. obtained the entire morphology of footprints under physiological conditions using QI (quantitative imaging) mode, which records the FD curve at each pixel, making it possible to obtain information on the morphology and mechanical properties simultaneously.^65,66^ As depicted in Figure 4(d), the footprint is oval-shaped with protein depletion in the central region, and the entire footprint is approximately 20–40 μm in diameter. Vancso’s team utilized AFM to observe the footprints left by cyprids walking on two modified surfaces (R–NH_2_- and R–CH_3_-terminated glass surfaces).^67^ They suggested that the deposited footprints had a fibrous appearance (Figure 4(e)), which may provide mechanical toughness to enhance the adhesive’s ability to resist deformation under seawater shear forces. Furthermore, they obtained a more microscopic high-resolution image of the footprint, revealing isolated chains and bundles of protein aggregates within the network structure of the footprints (as shown in Figure 4(f).^68^ Additionally, this group found that the footprint size and the microsized fiber thickness on CH_3_-glass were larger than those on NH_2_-glass, indicating different conformations of footprint proteins via reorganization and self-assembly.^69^ As for marine bacteria, Malfatti et al. studied the shapes, surface morphology, and size distribution of pelagic bacteria using AFM (Figure 4(g)).^70^ In another study, the swelling gel-like structure of adhesives secreted by green algae spores was observed in contact mode, revealing the natural hydrated state of the adhesive pads (see Figure 4(h)).^71^ Lastly, the mussel adhesive protein deposited on mica, as shown in Figure 4(i),^25^ exhibits a homogeneous distribution. This distribution attests to its strong adhesive properties and provides a reliable foundation for subsequent adhesion measurements. These examples highlight the significant role that AFM plays in elucidating the intricate morphologies of bioadhesives across various fouling organisms.

## How Does AFM Help to Characterize and Understand the Mechanical Properties of Natural Adhesives?

We briefly mentioned earlier that forces operating between the AFM probe tip and samples can be quantitatively obtained for contact and noncontact situations. Measuring the forces acting on adhesive layers and their impact on adhesive performance is pivotal for understanding binding. As mentioned, AFM can characterize the structure and properties of materials across the length scales. So, the question arises: how can one utilize this characterization potential for bioadhesives? We also mentioned that we shall not introduce or describe the various imaging and probing modes of AFM, as there are excellent reviews on this subject in the literature. Here, we just summarize some major conclusions relevant to our subject.

**Figure 5:**
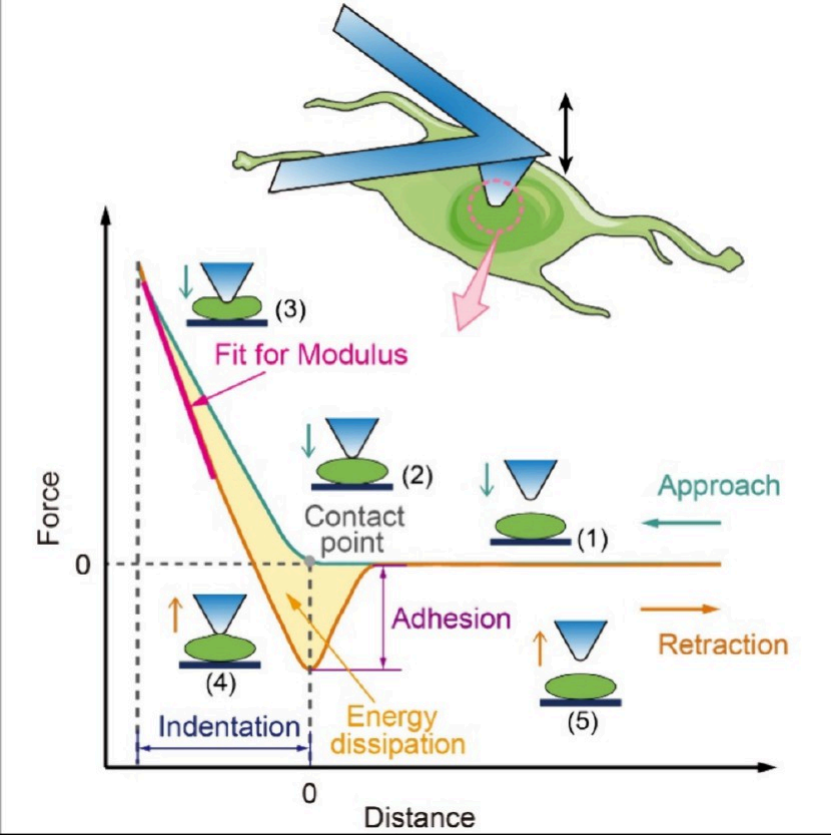
Schematic of AFM indentation on a soft cell and the corresponding FD curves. Cartoons depict the relative positions of the probe and the cell during different critical stages of the measurement process, as follows: (1) noncontact, (2) initial contact, (3) probe indentation into the cell, (4) adhesion in the retraction process, and (5) complete separation. Young’s modulus, adhesion force, and energy dissipation can be extracted from the retraction curve.

When in contact, the AFM tip can function as a nanoindentation probe. During experimental cycles, the AFM probe indents into the sample surface with controlled force, generating a force–distance (FD) curve reflecting the sample’s deformation response (see Figure 5). Various nanomechanical properties can be elucidated through the analysis of such FD curves, which provide insights into the indentation, elastic modulus, adhesion, and energy dissipation, as shown in Figure 5.^29^ Indentation refers to the depth to which the AFM tip penetrates the sample surface, which can be used to assess the hardness and compressibility of the sample. Young’s modulus is calculated from the initial linear region of the retraction curve by fitting it to a suitable model (e.g., Hertz, DMT, and JKR models).^39^ The adhesion force can be measured as the maximum force in the retraction curve.^72^ The energy dissipation (which represents the energy loss caused by an irreversible process) is determined by the hysteresis between approach and retraction (yellow shaded area in Figure 5).^72^

By employing FD experiments, understanding the nanomechanical properties of fouling organisms and their adhesive characteristics has been significantly enhanced. For example, Callow et al., through AFM indentation experiments, measured the adhesion strength (∼173 mN/m) and Young’s modulus (∼0.54 × 10^6^ N/m^2^) of the freshly released adhesive from the green algal spore.^71^ They observed a decrease in adhesion strength and an increase in Young’s modulus as the adhesive cured, as these changes could be directly observed over time in the FD curves. These changes reflect the adhesive’s transition from a viscous state to a harder, more solid material, providing evidence of cross-linking and hardening of the adhesive. Similar trends in barnacle cement and mussel adhesive plaques suggest a common strategy among marine biofoulers to secure long-term adhesion through adhesive hardening.^53,73^

Wetherbee et al. identified two unique mucilage layers on live diatom surfaces. The superficial mucilage layer displayed an adhesive force of 3.5 nN, while adhesive strands secreting from the raphe could withstand rupture forces up to 60 nN.^74^ These changes are considered pivotal to both adhesion and motility. Walker’s team investigated Young’s modulus of barnacle cement in air and discovered that the modulus increased from the outer layer to the inner layer.^75^ Similarly, *Ulva* adhesive exhibited a multilayered structure in its elastic and viscoelastic properties, with Young’s modulus increasing from the outer to the inner layers.^76^ The softer outer layers potentially provide the necessary flexibility for initial adhesion, enabling adaptation to irregular surfaces and filling of minute gaps. In contrast, the cross-linking internal layers enhance cohesive strength and resistance to shear forces, thereby contributing to improved durability and stability of the attachment.^76^

Poddar et al. further underscored the environmental sensitivity of bioadhesive mechanics by conducting real-time monitoring of a marine bacterium exposed to Co^2+^ ions. They demonstrated how bacteria can dynamically adjust their adhesion strength in response to changing chemical environments.^77^ Additionally, Abu-Lail and Camesano found that the adhesion force between bacterial surface biopolymers and the AFM tip increases with high salt concentrations, likely due to a conformational change in the biopolymers that enhances their adhesive properties.^78^

In summary, AFM-derived insights provide a profound understanding of marine biofouling’s complex, adaptive mechanisms. The curing behaviors, multilayered structures, and environmental sensitivity exhibited by natural adhesives are a testament to their sophisticated evolution in the challenging marine environment, which enables robust adhesion even amidst variable conditions. The ability of AFM to reveal such intricate details contributes to the design of biomimetic adhesives and effectively informs strategies for combat biofouling.

With the advancement of the method, AFM has become an enabling tool for high-resolution quantification and mapping of mechanical properties through arrays of FD curves or parametric methods.^29^ Initially, mechanical mapping featured limitations, such as slow imaging speeds, arbitrary modulation frequencies, and difficulty mapping heterogeneous surface elasticity.^79^ To overcome these obstacles, parametric nanomechanical methods such as bimodal AFM and contact resonance AFM have been developed for the *in situ* detection of various components of soft matter, including proteins, cells, and polymers.^80,81^ For example, Galluzzi et al. recently advanced the development of nanomechanical characterization of heterogeneous soft matter using finite element simulations based on AFM data.^82^ It is conceivable that with further advancement of the technique, the use of various mechanical mapping may open the way to further understanding the links among mechanical response, morphology, and function of natural adhesives.

## Measurements of Adhesive Forces by AFM Colloidal Probes: Mesoscale Studies

### AFM Colloidal Probes

AFM colloidal probes are invaluable for mesoscale studies, offering a complementary advantage over traditional probes for measuring adhesive forces. Colloidal probes consist of a spherical particle attached to the apex of a tipless cantilever, which typically has a radius of curvature in the micrometer to tens of micrometers range.^83^ The well-defined spherical tip of the colloidal probe provides a consistent interaction area that can be characterized, facilitating more accurate modeling and analysis.^84^ The larger tip radius results in a higher signal-to-noise ratio, which is beneficial for studies where subtle interactions are interesting. On the other hand, averaging interactions over a larger area means that ensemble averages are determined instead of isolated molecular events. Additionally, colloidal probes take into account the average interaction forces across multiple asperities on rough surfaces, providing a more comprehensive understanding of the adhesive behavior at the ensemble level.^85^

Several methodologies have emerged for securely attaching smooth microspheres to the tip-free end of the cantilever. Typically, a trace amount of glue (e.g., epoxy resin, UV-curable glue) is placed on the cantilever to fix the microsphere. This process can be realized by dipping a cantilever in glue and then attaching a microsphere.^86^ Alternatively, high-temperature sintering has been implemented to mitigate potential contamination from adhesive dissolution in liquids.^85^ In this procedure, a microsphere is initially adhered to a glycerol-coated cantilever. Upon heating, the glycerol evaporates, resulting in the microsphere being firmly attached after annealing. This technique is suitable for materials with melting temperatures below the melting of the cantilever, such as polystyrene and borosilicate glass, compared to silicon nitride or silicon cantilevers (1200–1900 °C). Finally, Vorholt et al. introduced a modular AFM method that enables the reversible immobilization of functionalized silica beads by applying negative pressure to a microchanneled cantilever.^87^ Given a recent review discussing the applications of colloidal probes to image and study marine fouling,^88^ we focus primarily on AFM-related research, i.e. probing surfaces at the nanometer scale. However, in the subsequent subsection, we provide a brief account of some studies using colloidal probes, to complement AFM results.

### Applications of Probing Protein–Surface Adhesion Forces at the Mesoscale Employing Colloidal Probes

**Figure 6:**
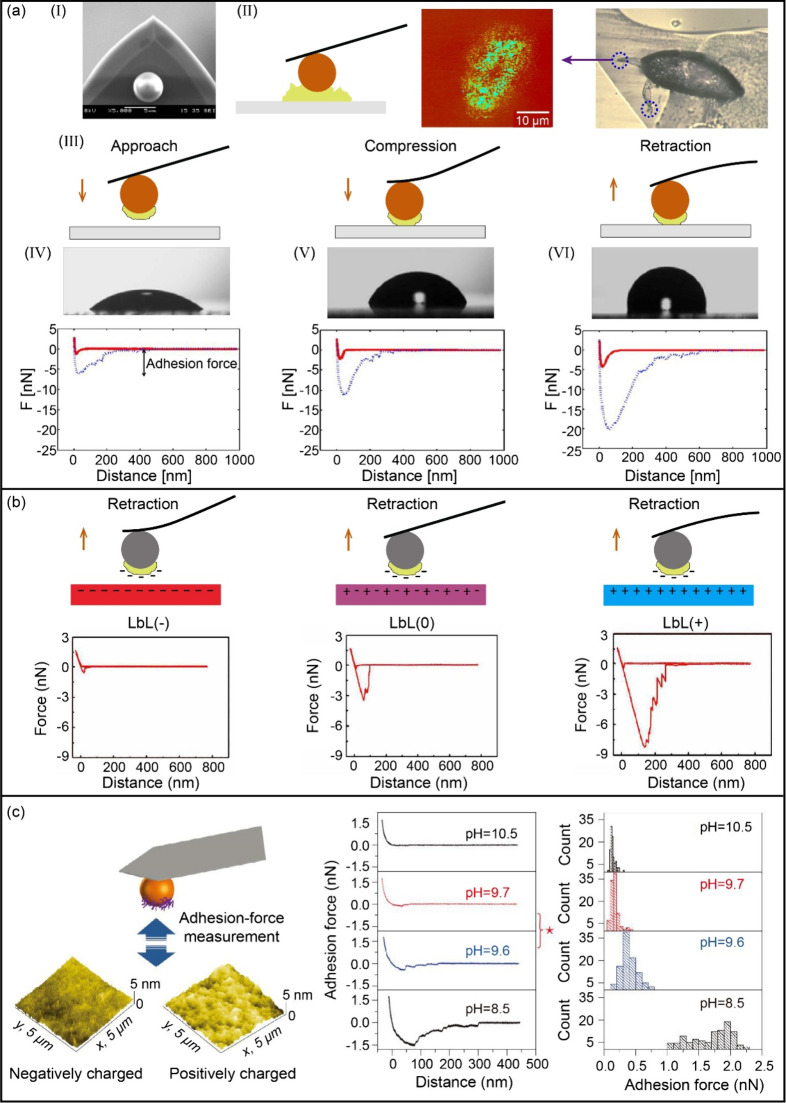
Schematic of measuring the adhesion force between proteins and various surfaces using colloidal probes. (a) Measurement of adhesion between cyprid footprints and surfaces with different wettability. (I) SEM image of the colloidal probe. (II) Probe modification with footprint proteins. (III) Probe-substrate interaction during force curve measurements. (IV) Force curve of footprints with low contact angle surface (40°), (V) with medium contact angle surface (65°), and (VI) with high contact angle surface (95°). Adapted from ref (65). Copyright 2014 American Chemical Society. (b) Measurement of adhesion between Bovine Serum Albumin (BSA) and LbL films with negative, zero, and positive potential, respectively. Reprinted from ref (90). Copyright 2016 American Chemical Society. (c) Measurement of the interaction of cyprid footprint-modified probe and charged surface under different pH conditions. The red star represents a significant change in adhesion force and indicates the pI of proteins. Adapted with permission from ref (92). Copyright 2016 Springer Nature.

In addition to being used for high-resolution nanomechanical and topographical imaging of biological materials such as living cells,^89^ colloidal probes are also extensively used in interfacial force measurements. In marine fouling studies, colloidal probes covalently attached by cyprid footprints using glutaraldehyde have been effectively employed to detect the adhesive strength between footprint proteins and various surfaces in seawater. Guo et al. demonstrated that the adhesion forces of footprints on hydrophobic surfaces were higher than on hydrophilic surfaces by creating a series of surfaces where wettability was the sole variable (see Figure 6(a)), suggesting that hydrogen bonding plays a less significant role in adhesion compared to hydrophobic interactions.^65^ Expanding on this methodology, they further investigated the adhesion forces between adsorbed proteins and layer-by-layer assembled films with varying surface potentials. As shown in Figure 6(b), the results indicated the strong adhesion between the protein and the surface with an opposite charge, suggesting that the electrostatic interaction was the driving force for protein adsorption.^90^ Most recently, the authors reported the adhesive strength between footprint proteins and zwitterionic polymer films, attributing the low adhesion force of the methacrylamide-based brushes to enhanced hydration.^91^ Colloidal probes have also been applied to measure the adhesion between proteins and charged surfaces under varying pH conditions (Figure 6(c)).^92^ More importantly, these measured proteins’ isoelectric point (pI) values can be estimated from the FD curves. With this method, the pI of footprint protein was determined to be in the range of 9.6 to 9.7, demonstrating the feasibility of measuring pI values even for trace amounts of protein. Using colloidal probes, Hu et al. reported the self-assembly behavior and adhesion properties of rBalcp19k, a recombinant barnacle cement protein.^51^ Bremmell et al. employed colloidal probes to study the interactions between fibrinogen and PEG-like plasma polymer surfaces.^93^ They observed that the adhesion disappeared when the measurement medium was changed from buffer to water, suggesting a transition in molecular conformation from extended in the buffer to more compact in water. Additionally, by attaching native bacterial cells to glass beads, Lower et al. measured their interfacial and adhesion forces with the mineral surfaces *in situ*, contributing to elucidating the interactive dynamics between living bacteria and mineral surfaces.^94^ We note that beyond AFM colloidal probes, the surface force apparatus (SFA) has also been utilized to measure the adhesion of mussel adhesive proteins to interfaces and the cohesion between proteins.^25,95,96^ A detailed technical description of this method was reported by Israelachvili et al.^97^ Recent studies have highlighted the complementary nature of SFA and AFM in probing bioadhesive interactions.^98,99^ Let us comment once more on the role of the interaction area in the information delivered. Both SFA and colloidal probes offer insights at the mesoscale level, in which the contact area in SFA measurements is usually 10^–3^ mm^2^ and that of a colloidal probe is about 10^–12^ mm^2^.^100^ Thus, clearly, ensemble properties are delivered. Measurement at such mesoscale levels helps understand the adhesion of various adhesive proteins, but it is insufficient to elucidate the molecular mechanism of adhesion.^101^ One of the significant advantages of AFM (as opposed to SFA and colloid probes) is that in addition to structure imaging with a resolution down to the nanoscale (including images of proteins), it can also be used to measure forces at the single molecule level and perform force spectroscopy measurements.^98^ More specific details of using AFM to explore molecular adhesives are covered in the following paragraphs.

## Expanding the Horizons of AFM for Interaction Studies at the Molecular Scale

### AFM-Based Single Molecule Force Spectroscopy

For many years, studies of the adhesion of marine fouling organisms have encompassed primarily ensemble-level investigations. Thus, the underlying physical and chemical molecular principles that govern the interaction of adhesion proteins with specific surfaces remained elusive. At the molecular level, however, active proteins are anticipated to regulate the adhesion behavior since many proteins can sense the external environment and change conformation accordingly.^102^ Therefore, a molecular-level understanding of how surface properties affect the kinetics of protein adsorption and protein conformation changes will further guide the understanding and the design of effective and nontoxic marine antifouling coatings. The advent of AFM-based single-molecule force spectroscopy provides new possibilities for the analysis of intermolecular interactions, including protein–protein and protein–substrate interactions,^103,104^ and of single-chain synthetic polymers.^105^

Before we move on to discussing the results of AFM single molecule force spectroscopy (AFM-SMFS), we briefly make a few comments about some technical details. In short, isolated molecules are attached to the probe tip, and their interactions, including adhesion to the substrate or another molecule immobilized on the substrate, are monitored.^106,107^ This approach allows quantitative measurements of the forces required to separate a molecule from a specific surface, providing valuable information for exploring the interactions of adhesion proteins with different surfaces and bond strength. Protein unfolding processes also carry a characteristic fingerprint to the conformational changes that occur during stretching. Mechanical denaturing and unfolding pathways can thus be studied in molecular detail.^108^

### Modification of AFM Probes

**Figure 7:**
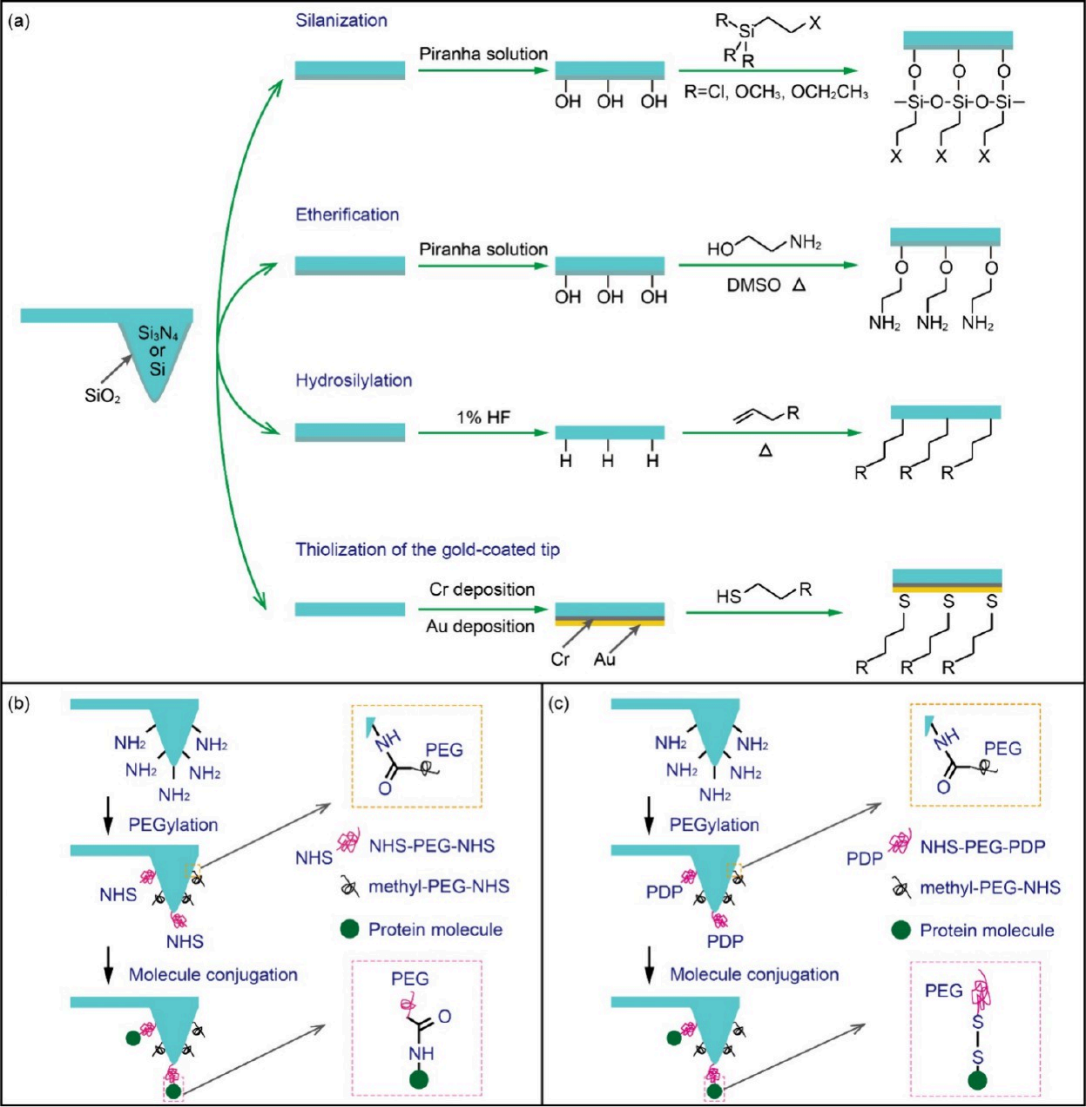
Schematic representation of AFM tip chemical functionalization approaches. (a) Examples of attachment strategies. (b, c) Schematic showing the coupling of proteins to a silicon tip using (b) NHS-PEG-NHS and (c) NHS-PEG-PDP linkers.

In the realm of single-molecule studies, the chemical modification of AFM probes has emerged as a pivotal technique, providing unprecedented access to measuring the elasticity of single polymer chains, conformational changes, desorption kinetics of surface-bound macromolecules, host–guest interactions, and even the manipulation and delivery of single molecules.^109−111^ We provide a schematic in Figure 7, to illustrate representative examples of tip-functionalization approaches, and then discuss these in the subsequent section.

In single-molecule force spectroscopy experiments, biomolecules are typically immobilized on AFM tips or substrates using physical adsorption or chemical bonding. Gaub et al. exemplify the utilization of physical adsorption by attaching avidin to the silicon nitride surface of the AFM tip to explore its interaction with biotin.^109^ Despite its simplicity, physical adsorption often leads to nonspecific and multiple attachments, precluding the observation of unambiguous single-molecule behavior and accurate measurement of binding strengths.^26^ To overcome this limitation, alternative covalent bonding methods are adopted, using flexible bifunctional cross-linkers. In this process, one end of the cross-linker is first anchored to a functionalized tip or substrate, followed by the attachment of the target molecule to the other end. Commonly employed linkers include short polyethylene glycol (PEG) and oligomers, with PEG being the most widely used due to its versatile synthesis, tunable chain lengths, and functionalizable termini.^106^

The prevalent approach for modifying AFM probes involves the chemical treatment of commercially available tip-cantilever systems, often by creating self-assembled monolayers (SAMs). Silicon or silicon nitride probes are initially activated in a piranha solution (H_2_SO_4_: 30% H_2_O_2_ = 7:3, v/v) to expose the silanol group (Si–OH) for subsequent modifications.^112^ As shown in Figure 7(a), after activation, further chemical modifications of these exposed silanol functionalities can be achieved through silanization, etherification, and hydrosilylation. Alternatively, leveraging the strong affinity between gold and thiol groups (Au–S), alkanethiols can spontaneously assemble into a closely packed, well-ordered monolayer on the gold surface.^113^ Silanization is a popular surface modification method involving the reaction of silanol groups with silane solutions (e.g., alkylchlorosilanes or alkylalkoxysilanes) to form an organosilane monolayer.^114^ However, employing multifunctional silanes, especially those with three reactive ends, poses the risk of undesirable polymerization reactions and hydrolytic instability.^115^ Etherification, an alternative method, uses ethanolamine to react with silanol groups, resulting in an amino-terminated (−NH_2_) organic monolayer.^116^ During hydrosilylation treatment, the activated silicon surface is treated with hydrofluoric acid (HF) to generate hydrogen-terminated silicon (Si–H) sites. This is followed by a reaction with 1-alkenes to form alkane monolayers via robust covalent Si–C bonds.^117^ Regarding gold-coated AFM tips, a common practice is depositing a thin chromium (Cr) layer before gold coating to enhance adhesion, followed by direct thiolation for functionalization.^26^

Following the modification process described above, AFM probes are often subjected to further cross-linking with flexible cross-linkers to enhance their functionality for single-molecule studies. As an illustrative example, Figure 7(b) depicts the coupling of proteins to an amino-functionalized tip using bifunctional PEG. Initially, the probe’s surface is functionalized with amino groups, which are then immersed in a solution containing *N*-hydroxysuccinimide (NHS) activated PEG derivatives (NHS-PEG-NHS and methyl-PEG-NHS). The NHS ester groups react with the surface-bound amino groups, forming stable amide bonds that tether PEG chains to the probe’s surface. This step effectively bridges the probe with PEG linkers, which act as adaptable spacers that can accommodate a wide range of biomolecules while preserving their native conformation.^35^ Following the PEGylation process, the probe is immersed in a solution containing the target protein molecules. The NHS ester termini present on the NHS-PEG-NHS linker can again react with the amine groups of the proteins, thus facilitating the covalent attachment of proteins to the probe’s surface. Additionally, as shown in Figure 7(c), proteins can also be irreversibly bound to the probe via disulfide bond formation between the reactive 2-pyridyldithiopropionyl (PDP) group and the free thiols presented by cysteines in the protein. This conjugation technique allows for controlled and oriented immobilization of proteins.^35^ Furthermore, methyl-terminated PEG segments serve as an additional layer of protection. The methyl group provides steric hindrance that minimizes nonspecific interactions between the probe and the sample. Ultimately, this dual-functionalization strategy enhances the probability of detecting single molecular events by reducing background noise caused by nonspecific binding, thus improving the sensitivity and specificity of AFM-based studies in biofouling research and related applications. Comprehensive methodologies for AFM tip modification are detailed in ref.^118^

### Single-Molecule Recognition

**Figure 8:**
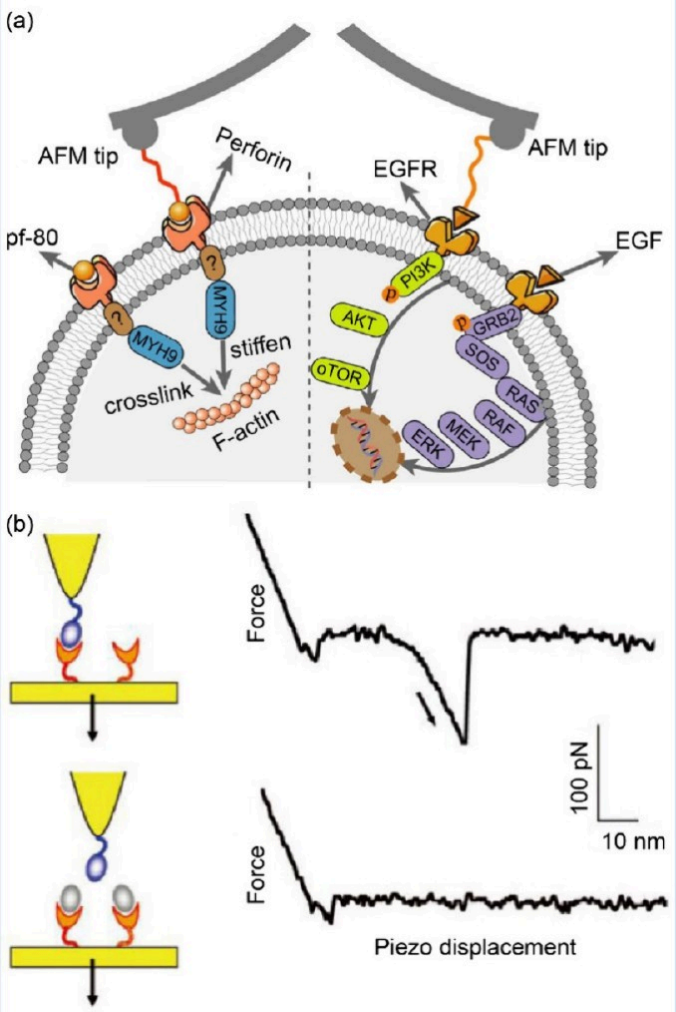
Schematic diagram of molecular recognition. (a) The specific recognition and detection of the molecule related to cell death by single-molecule force spectroscopy technique. Reprinted from ref (121). Available under a Creative Commons CC-BY 4.0 License. Copyright 2024 The Authors. Published by Walter de Gruyter GmbH. (b) Measurement of molecular recognition interaction forces. Reprinted with permission from ref (26). Copyright 2006 Springer Nature.

AFM-SMFS is a technique that enables the specific identification and detection of individual molecules (Figure 8(a)). The adhesion force observed in FD curves is a critical metric that reflects the unbinding force between complementary receptor and ligand molecules, which is central to molecular recognition (Figure 8(b)). However, interpreting these curves is not straightforward, as they can exhibit complex behavior due to the interplay of various factors. In FD curves, the initial force peak often corresponds to the rupture of nonspecific adhesion, which can occur when the AFM tip contacts the sample surface. Following this, if the interacting molecular pairs are stiff and provide a stable contact, subsequent force peaks may represent the rupture of these specific interactions. The presence of flexible molecules, such as long biomolecules or cross-linkers, introduces an additional layer of complexity. These molecules can undergo elongation before the unbinding event, leading to a force curve with multiple peaks.^26^ Upon further extension, stretching these flexible molecules (which could involve more than one interaction) leads to the breaking of the weakest point of contact.^119^ Moreover, the rupture forces can be influenced by the force loading speed that the molecule experiences, which is the rate at which the cantilever is retracted from the sample.^28^ Measuring the rupture forces at different unloading speeds can provide information about a bond’s kinetic properties and potential energy landscape.^120^

Ideally, the contact area of the AFM tip or surface could be assigned to a single molecule, thereby avoiding the stretching of multiple bonds. However, achieving precise control over the modification of a single molecule on a surface and assuring that there is only one single connecting molecule remains a significant technical challenge. One strategy to minimize the multiple molecule interactions during single-molecule measurements is to graft in a dilute molecular solution, which ensures a low surface density of molecules on the tip surface.^118^ In such cases, clustering should be considered as this could perturb the required even distribution of the surface-attached molecules.

Investigators have also utilized statistical methods to extract meaningful data from large data sets of FD curves. By constructing histograms of pull-off force values from hundreds to thousands of FD curves, they can apply Gaussian statistics to identify the most probable force corresponding to a specific molecule interaction. This then allows the determination of single molecule bonding strength values and other parameters of the bond interaction energy landscape, such as energy barrier and unbinding distance.^122,123^ Furthermore, the analysis of histograms of rupture forces has enabled the separation of specific binding interactions from nonspecific interactions, providing a clearer picture of the molecular recognition process.^124^ We can conclude that advances in AFM probe preparation and functionalization techniques continue to refine our ability to interrogate molecular recognition at the highest resolution, thereby expanding the horizons of AFM for interaction studies down to the molecular level.

### AFM-Based Force Spectroscopy of Natural Adhesives

AFM-based force spectroscopy was introduced in the previous section, which discusses tip functionalization approaches and the lessons one can learn from such measurements. Now, we shall focus on applications to enhance the understanding and applications of molecular adhesion relevant to marine fouling.

**Figure 9:**
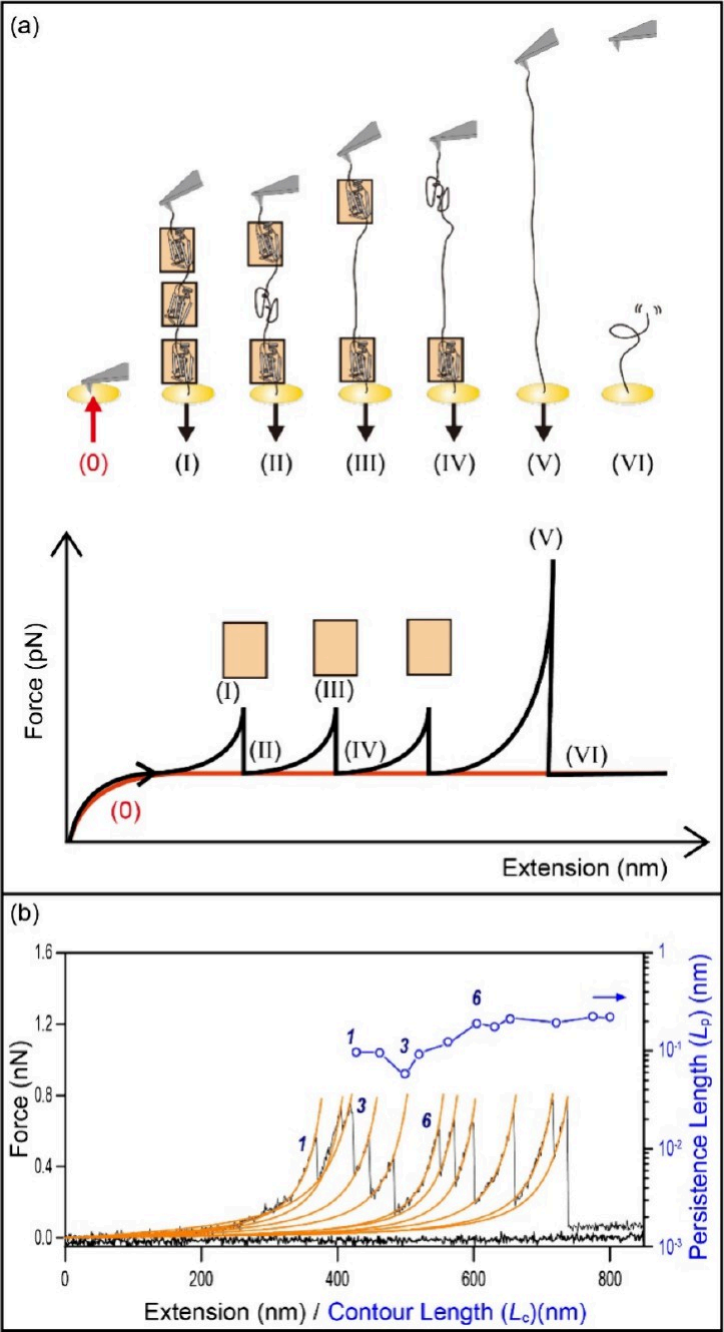
Schematic of polyprotein unfolding force spectroscopy and WLC model fitting. (a) Upper: The process of the AFM tip stretching the polyprotein at a constant velocity. The protein here has three domains. Lower: The corresponding force–extension line tracing of the stretching process. The red arrow represents the tip approaching the substrate, and the black arrow indicates the retraction of the tip. (0) ∼ (VI) represents typical states of the stretched protein and the related force spectroscopy data. Reprinted with permission from ref (132). Copyright 2016 IOP Publishing Ltd. (b) Description of sawtooth unfolding peaks using the WLC model (orange lines) and the obtained contour length (*L*_C_, circles) plot versus persistence length (*L*_P_). Reprinted with permission from ref (52). Copyright 2010 Royal Society.

Significant strides have been taken in harnessing AFM-based force spectroscopy to elucidate the nanomechanical attributes and adhesion mechanisms of natural adhesives, offering novel perspectives on their functionality.^106,125^ Early pioneering work by Smith et al. in 1999 unveiled a hallmark characteristic in the force–extension profiles of natural adhesives–a distinctive sawtooth pattern.^126^ Notably, further research by Hongbin Li et al. focused on the extensibility of the protein titin, which similarly exhibited this characteristic sawtooth pattern in force–extension measurements.^127^ This pattern was ascribed to the ordered, sequential unfolding of titin’s constituent immunoglobulin domains under tensile force, providing valuable insights into the protein’s complex elasticity. As depicted in (Figure 9(a)), this sawtooth pattern is emblematic of the step-by-step unfolding and unbinding of compactly folded protein fibers when subjected to tensile stress. Each tooth in the sawtooth profile symbolizes the unfolding of a single protein domain, leading to a stair-step reduction in the applied force as the protein progressively unfolds. This modular unbinding mechanism confers remarkable toughness and elasticity to the adhesive material, as each unfolding event dissipates energy, enabling the material to withstand substantial deformation before reaching a critical failure threshold. Subsequent research has observed analogous mechanical signatures in the adhesive secretions produced by diverse marine fouling species, such as diatom,^128^ green algae,^75^ and barnacle cyprids.^52^ The toughness of these materials has been attributed to the hidden length and sacrificial bonds, which consumed extra energy during the unfolding of the tertiary structure of the proteins and the breaking of sacrificial bonds.^129^

When a polymer chain undergoes stretching, it encounters the influence of entropic and enthalpic forces that define its mechanical response. Entropic elasticity prevails at modest extensions, driven by the reduction in the chain’s configurational entropy as the unconstrained random coil conformation is constrained. This effect is primarily due to the restricted freedom of the chain segments to adopt multiple conformations upon elongation.^130^ However, enthalpic contributions become significant as the extension increases beyond a certain threshold relative to the chain’s contour length.^131^ The covalent bonds and secondary structures within the polymer backbone experience direct tension, leading to a rise in enthalpic elasticity that results from the deformation and potential rupture of these chemical bonds. Statistical mechanical models, such as the Freely Jointed Chain (FJC) and the Worm-Like Chain (WLC) models, are pivotal tools for interpreting the complex force–extension data acquired during stretching experiments. The WLC model has proven remarkably versatile, accounting for the elastic restoring force exerted by a polymer chain in response to the diminishment of its conformational space.^76^ This model has been extensively adopted for fitting the characteristic sawtooth force–extension profiles observed in experimental data, which reflect the stepwise unfolding and unbinding events along the polymer chain. The WLC model is suitable for single-chain stretching and for stretching parallel-chain segments, which is the most commonly encountered situation in experiments. Despite its widespread and successful application, it is crucial to note that a critical aspect of the analysis involves accurately determining the “zero point” in the force curves. Accurate zero point determination is essential for the reliable interpretation of the force–extension data and the subsequent extraction of meaningful parameters. As shown in Figure 9(b), using the WLC model, two parameters, i.e.values of contour length and persistence length can be obtained. The former represents the maximum extension length of the polymer, and the latter characterizes the stiffness of the molecular chain.^132^ These parameters afford valuable insights into the nanoscale mechanics of natural adhesives and contribute to a comprehensive understanding of their formidable adhesion capabilities.

**Figure 10:**
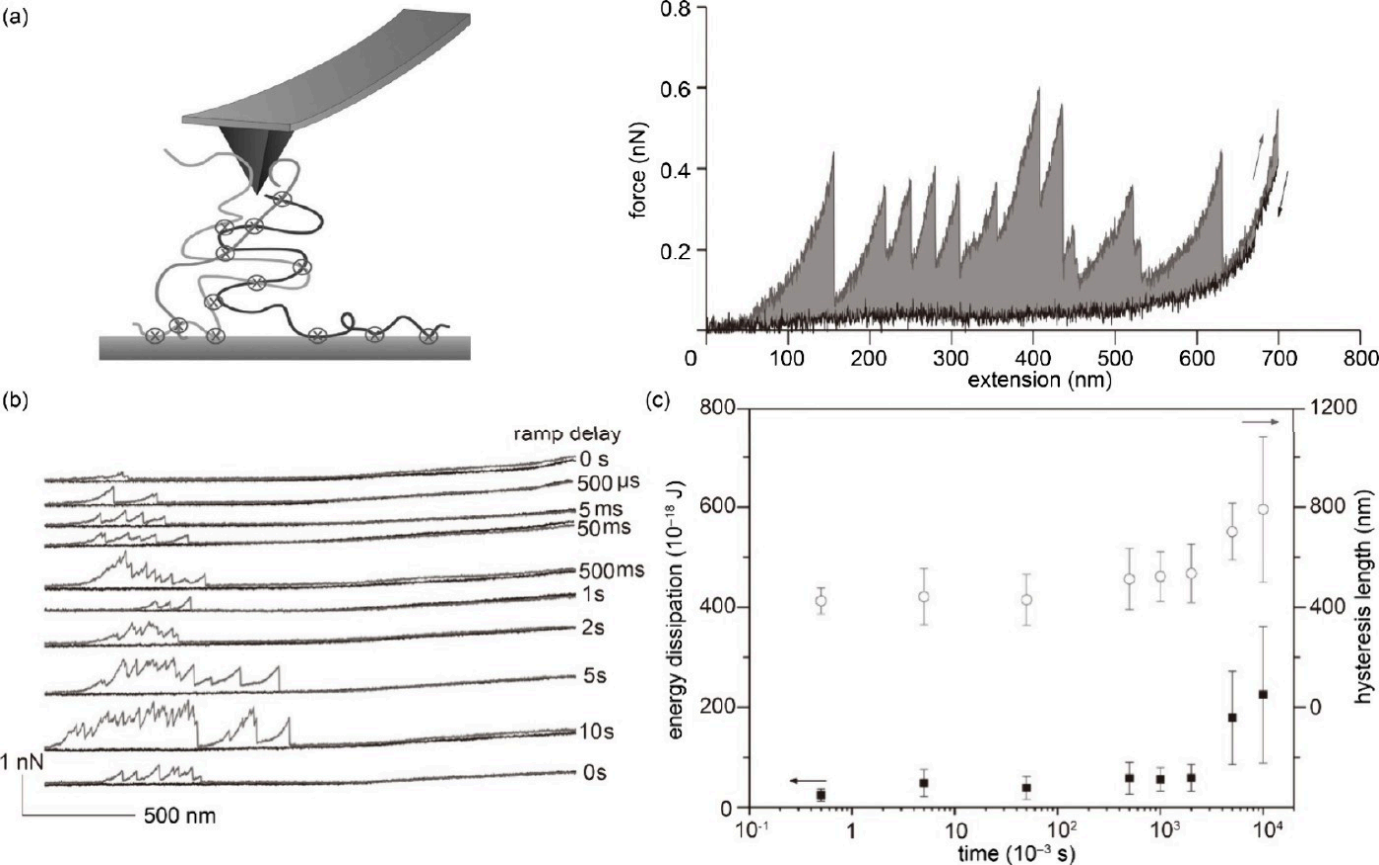
Nanomechanical properties of cyprid footprints by AFM force spectroscopy. (a) Left: The pickup of footprint molecules interconnected sacrificial bonds (represented by crosses) by an AFM tip. Right: Force spectra recorded from a footprint of a barnacle cyprid. The mechanical unfolding of footprint nanofibrils shows a sawtooth characteristic with multiple progressive unfolding peaks and monotonic entropic elasticity in the relaxation curve. (b) The refolding dynamics of nanofibrils allowed different relaxation times (delay time from 0 to 10 s). (c) The dissipation and hysteresis length were calculated from the force–extension curves with varying delay times. Adapted with permission from ref (52). Copyright 2010 Royal Society.

Sawtooth-like AFM force curves obtained on diatom (*Toxarium undulatum*) adhesives (“mucilage pad”) were obtained and fitted by Wetherbee et al. using the “wormlike chain” model.^133^ The numerical evaluation resulted in average rupture force values of 794 pN and a persistence length of about 0.026 nm. Notably, they employed a technique metaphorically called “fly fishing,” wherein the AFM tip is raised and lowered like a fisherman casting a line, hoping to engage and manipulate a single polymer chain selectively. The rupture force value obtained through this “fly-fishing” technique is notably larger than that reported for the previously studied species *Craspedostauros australis* (197 pN),^128^ which may be attributed to the diatom adhesive studied here being a cohesive unit composed of a series of modular protein molecules that form connecting nanofibers being stretched. Furthermore, when the adhesive nanofibers were subjected to repeated stretching and relaxation (up to several hundred consecutive cycles), accurate overlapping sawtooth curves were obtained, demonstrating the bond rupture’s reversible nature.^133^ Regarding the footprint secreted by barnacle cyprids, Vancso’s team proposed that when “sacrificial bonds” are broken during stretching, the hidden polypeptide chain of the protein unfolds, dissipating energy and resulting in a sharp drop in force (Figure 10(a)).^52^ We note that the concept of “sacrificial bonds” has been introduced by Hansma et al. to interpret the fracture toughness of biomaterials, particularly emphasizing its role in bone, where sacrificial chains provide a reversible energy-dissipation mechanism, enhancing their toughness.^129^

The authors determined that the rupture forces of the sacrificial bonds from 220 to 580 pN, and the energy required to break the sacrificial bonds is about 120 × 10^–18^ J. Repeated unfolding peaks observed during the stretching experiment manifested that the footprint protein domains can be reformed within 5 s in the relaxed-stage (Figure 10(b), (c)). This behavior provides a dynamic binding mechanism that helps resist hydrodynamic shear in the ocean, e.g., experienced by the impact of periodic waves in shore regions. While this work provides a valuable model for studying the properties and function of reversible adhesion of footprint proteins, several protein chains were inevitably stretched simultaneously in the AFM experiments. Additionally, to investigate the effect of wettability on the nanomechanical properties of natural adhesives, AFM tips were modified with different functional groups (either hydrophilic or hydrophobic), and used in the tensile test. With this method, Vancso’s team reported that footprint proteins exhibit a greater affinity to the hydrophobic tip.^68^

**Figure 11:**
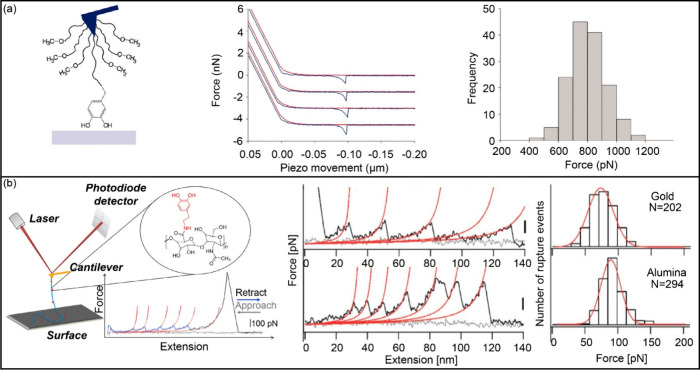
Typical application of force spectroscopy in measuring adhesion force. (a) Left: DOPA adheres firmly and reversibly to Ti surfaces. Middle: The four different force curves were produced from the same DOPA-functionalized tip and are displaced vertically for clarity. Right: Histogram (*n* = 147) of pull-off force values for DOPA-Ti obtained with a single AFM tip at a loading rate of 60 nN/s. Adapted with permission from ref (134). Copyright 2006 National Academy of Sciences, U.S.A. (b) Left: Scheme of single molecule AFM experiments on DOPA-surface interactions using the “multi-fishhook” approach. DOPA molecules (highlighted in red) were conjugated to hyaluronan (HA, colored in cyan) polymer through amide bonds. The sawtooth-like curve in blue indicates rupture events between DOPA and the surface. Each peak in the curve corresponds to one such event. The middle panel shows representative force–distance curves for HA-DOPA rupture with different substrates at a pulling speed of 1000 nm/s. The histograms for rupture forces for the same substrates are shown in the right panel. Adapted from ref (135). Copyright 2014 American Chemical Society.

Intricate physicochemical interactions, such as van der Waals forces, hydrophobic interactions, electrostatic interactions, hydrogen bonding, and specific binding, drive the adhesion of microbes, live cells, and biomolecules. Single-molecule force spectroscopy has been successfully exploited to determine how these factors act on biological materials, making them adhere to each other or to a solid substrate. DOPA, the key adhesion protein in mussels, was previously introduced in part 2. Using PEG as a linker, Messersmith et al. decorated DOPA onto a Si_3_N_4_ probe to investigate the interactions of single-molecule DOPA residues and oxidized DOPA with organic and inorganic surfaces.^134^ They reported that the DOPA-Ti interaction is very strong reversible binding, reaching 800 pN (Figure 11(a)). However, the oxidation of DOPA dramatically reduced adhesion to Ti, while it can form covalent bonds to enhance adhesion to the organic surface. In a subsequent study, this team explored the adhesion of DOPA-containing peptides to organic and inorganic substrates. The results showed that increasing the peptide length enhances the adhesion strength, which indicates a promising avenue for optimizing the adhesive performance of bioinspired materials.^101^ Cao’s team introduced a “multi-fishhook” method, where multiple DOPA molecules were conjugated to a single hyaluronan (HA) polymer, enabling the rupture of numerous DOPA-surface bonds with each HA-DOPA pull (Figure 11(b)).^135^ This method facilitated high-throughput quantitative measurements of the interactions between DOPA and various wet surfaces. Subsequently, the authors quantitatively predicted the relationship between DOPA contents and binding strength based on the measured rupture kinetics. In recent work, this team quantified the cation-π interaction in mussel foot proteins-5 using single-molecular force spectroscopy. The results showed that individual cation-π interaction ruptures at about 70 pN, a strength comparable to that of other noncovalent interactions.^136^ Xu and Siedlecki evaluated the effect of surface wettability on protein adhesion to the surface of polymeric biomaterial by covalently cross-linking the proteins to the probes.^137^ Higher adhesion forces were observed on the hydrophobic surfaces compared to the hydrophilic surfaces. Reches et al. measured the interactions of five different amino acid residues with inorganic surfaces based on single molecule force spectroscopy and concluded that the electrostatic and hydrophobic interactions together determine the adsorption strength among amino acids and inorganic surfaces.^122^

In conclusion, AFM-based force spectroscopy played a pivotal role in elucidating the fundamental principles governing natural adhesives and in guiding the development of next-generation, bioinspired adhesive technologies.

## AFM Applications on Antifouling Coatings: Toward Knowledge Transformation

### Design of Antifouling Coatings Inspired by Adhesion Mechanism

**Figure 12:**
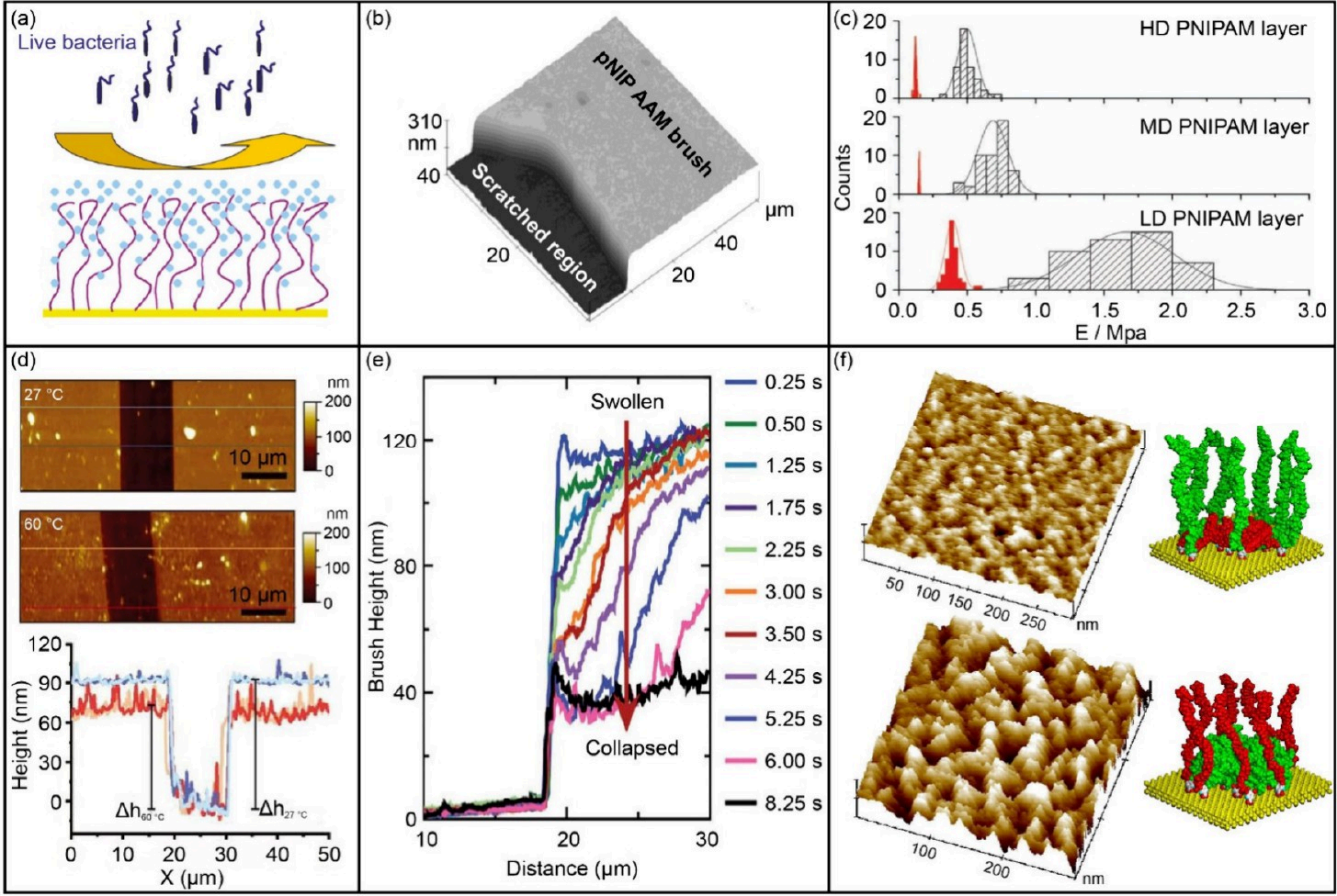
Schematic representation of the swollen brushes and examples of using AFM to study polymer brushes. (a) Swollen polymer brushes exhibit an antifouling property. Reprinted from ref (147). Copyright 2011 American Chemical Society. (b) Measurement of brush thickness. Reprinted from ref (144). Copyright 2004 American Chemical Society. (c) Statistical histograms of Young’s modulus of PNIPAM brushes in water (filled) and in water/methanol (50% v/v; patterned). Reprinted with permission from ref (150). Copyright 2011 Wiley-VCH Verlag GmbH & Co. KGaA. (d) Height images from tapping-mode AFM measurements of the thermoresponsive poly(NMEP) brushes in water at 27 °C (upper) and 60 °C (middle). The respective cross sections are displayed in the bottom image. Reprinted with permission from ref (152). Copyright 2022 John Wiley & Sons. (e) Sectional height analysis of a collapsing PMAA brush as the pH is switched from pH 10.5 to pH 3. Reprinted with permission from ref (153). Copyright 2009 Royal Society of Chemistry. (f) High-resolution tapping mode AFM topography images represented in 3D. Y2 in toluene (upper) and water (bottom) are shown here. Corresponding molecular models are shown as well. Reprinted with permission from ref (155). Copyright 2005 Wiley-VCH Verlag GmbH & Co. KGaA, Weinheim.

Among various antifouling methods, the application of polymer brushes as antifouling materials shows excellent potential and attracts increasing attention to studying how different brush characteristics affect the interactions with biofoulers. As a powerful tool for nanoscale imaging and mechanical characterization, it is not surprising that AFM has been widely exploited to investigate polymer brushes, including the imaging of surface morphology, the measurement of brush thickness, the estimation of grafting density, the observation of stimuli-responsive behavior, and evaluation of antifouling properties.^138,139^ In short, polymer brushes are composed of densely packed polymer chains that extend from a surface to create a brush-like conformation (see Figure 12 (a)).^140^ The preferred method for preparing polymer brushes is the “grafting from” technique, which allows for synthesizing thick and stable brushes with high grafting density.^141^ The physical and chemical characteristics of polymer brushes significantly influence their antifouling efficacy. Factors such as the length, density, and flexibility of the polymer chains and the surface morphology play crucial roles in determining the brush’s performance. Consequently, a thorough understanding of these structural parameters is essential for designing effective antifouling coatings. We believe that while brushes provide very useful platforms for understanding the relationships between marine fouling and surface characteristics, their large-scale technological application is challenged by the scalability issues of their preparation methods.

### Applications of AFM in the Characterization of Antifouling Coatings Utilizing Polymer Brushes

Many studies have compared the surface morphology and roughness of various polymer brushes before and after grafting. As an example, Chen et al. utilized surface-initiated atom transfer radical polymerization (SI-ATRP) to graft polymer brushes from poly(vinylidene fluoride) (PVDF), which showed enhanced resistance to protein adsorption.^142^ The authors observed that the root-mean-square surface roughness of the PVDF surface was reduced after modification, indicating the presence of a smooth polymer brush layer. This modification effectively increased the PVDF surface hydrophilicity, which diminished nonspecific protein adsorption. Guo et al. compared the morphology of five zwitterionic polymeric brushes and found that one of them showed slightly larger clusters.^143^ Nevertheless, all the polymer brush coatings have similar surface roughness values, all less than 1 nm. Using AFM to measure the thickness of the brush, a mainstream method is to analyze the cross-sectional difference of the height image taken at the boundary between brushed and brushless areas, as shown in Figure 12(b). In order to remove the brush layer, one approach is to carefully scrape the brush with a blade tip until the substrate is exposed,^144^ while another alternative is to directly prepare a surface with patterned brushes.^145^ Takahara et al. investigated the effects of salt concentration on the dimensions and properties of superhydrophilic polymer brushes with zwitterion side groups, specifically poly(2-methacryloyloxyethylphosphorylcholine) (PMPC) and poly[3-(*N*-2-methacryloyloxyethyl-*N*,*N*-dimethyl)ammonatopropanesulfonate] (PMAPS).^146^ They used AFM to determine the swollen thickness of the brushes and found that PMPC brush thickness was independent of salt concentration, while PMAPS brush thickness significantly increased with salt concentration. Yu et al. have reported a strong correlation between brush thickness and antifouling performance.^147^ The authors observed topological differences between thin and thick polymer brushes and found that the thickness of an effective antifouling brush was around 20–45 nm. This is attributed to the fact that the longer polymer chains reduce hydrogen-bonding interactions due to entanglement, leading to a less hydrated surface to resist protein adsorption. However, the shorter polymer chains may not form a dense hydration layer and, thus, more protein adsorption.

The grafting density, defined as the number of polymer chains per unit surface area, has also been shown to influence the antifouling performance of films.^148^ To precisely quantify this grafting density or deduce Young’s modulus of such brush coatings, researchers employ the steric interaction model, an analytical framework developed for polymeric brushes, to analyze FD curves.^149^ This model provides valuable insights into the nanoscale mechanical behavior and structural organization of the brush layers, directly relevant to their antifouling capabilities. Additionally, with the help of the colloidal probe, Young’s modulus of polymer brush can also be estimated from FD curves based on the Hertz model (Figure 12(c)).^150^ However, these methods become imprecise when polymer brushes are covered on a deformable substrate since the FD curves obtained provide information about both the brushes and substrate deformations. In a recent work by Sokolov et al., a model considering both brushes and substrate deformations was developed, enabling quantitative characterization of the grafting density, brush thickness, and Young’s modulus of the substrate.^151^ In a recent study, Guo et al. prepared zwitterionic polymer brushes exhibiting antifouling properties through SI-ATRP.^91^ The grafting density and nanomechanical properties of the brush were characterized by colloidal AFM-based force spectroscopy, which validated the feasibility of using a colloidal probe to study marine antifouling polymer brush.

The morphology and nanomechanical properties of some polymer films can be significantly altered by changing external stimuli such as temperature, pH, or ionic strength. AFM can monitor responsive behavior in situ. An example was given by Zuilhof et al., who synthesized tunable thermoresponsive poly(NMEP) brushes and examined the reversible changes in the morphology of the brushes with AFM.^152^ It was discovered that when the temperature was below the lower critical solution temperature (LCST), the polymer brush immersed in water adopted a highly swollen conformation, and the average height of the polymeric features exhibited a sharp increase as compared with a dry state, from 42 to 91 nm. By contrast, as shown in Figure 12 (d), above the LCST, the thermoresponsive conformational change of the brushes from extended to collapsed states reduced the brush thickness. The temperature-induced alterations in brush conformation directly impact the material’s antifouling performance. In the swollen state, the increased volume of the polymer chains creates a steric barrier that hinders the approach of biofouler to the substrate, thereby preventing their adhesion. Moreover, the hydrophilicity of the extended polymer chains further impedes the adsorption of macromolecules and microorganisms by reducing the attractive interactions. Zauscher et al. observed the pH- and ionic-induced changes in the height of polyelectrolyte brushes, providing insights into their tunable responsiveness.^145^ The average brush height could be repeatable and reversibly switched between 9 nm at pH 4 and 112 nm at pH 9, underscoring the potential for creating dynamic, environmentally sensitive antifouling coatings. Furthermore, Parnell et al. directly visualized the real-time swelling and collapse of polyelectrolyte brushes as the pH changed (Figure 12(e)).^153^ These findings highlight the capacity of polyelectrolyte brushes to adapt their conformation in response to changing marine conditions, thereby impeding the attachment of biofoulers. In a recent work by Wanless et al., the response of hydrophobic polyelectrolyte brushes to varying solution ionic strength and specific anion was revealed using colloidal AFM-based force spectroscopy, highlighting the importance of ionic interactions in marine fouling resistance.^154^ Tsukruk and co-workers obtained, by using *in situ* AFM measurements, the morphology and nanomechanical properties of amphiphilic polymer brushes, revealing their varying friction and wear properties upon exposure to different solvents, which is crucial for their performance in marine environments (Figure 12(f)).^155^ These studies provide valuable insights into the rational design of tunable responsive surfaces, directly relevant to developing effective marine antifouling polymer coatings.

### AFM Applications on Other Typical Antifouling Coatings: Advancing Material Development

AFM has proven instrumental in unraveling the intricacies of various antifouling coatings beyond those based on polymer brushes by providing comprehensive insights into their surface morphology, mechanical properties, self-healing behaviors, assembly process, and bonding strength. This section delves into AFM’s diverse roles and contributions in understanding and enhancing the performance of other typical antifouling coatings.

**Figure 13:**
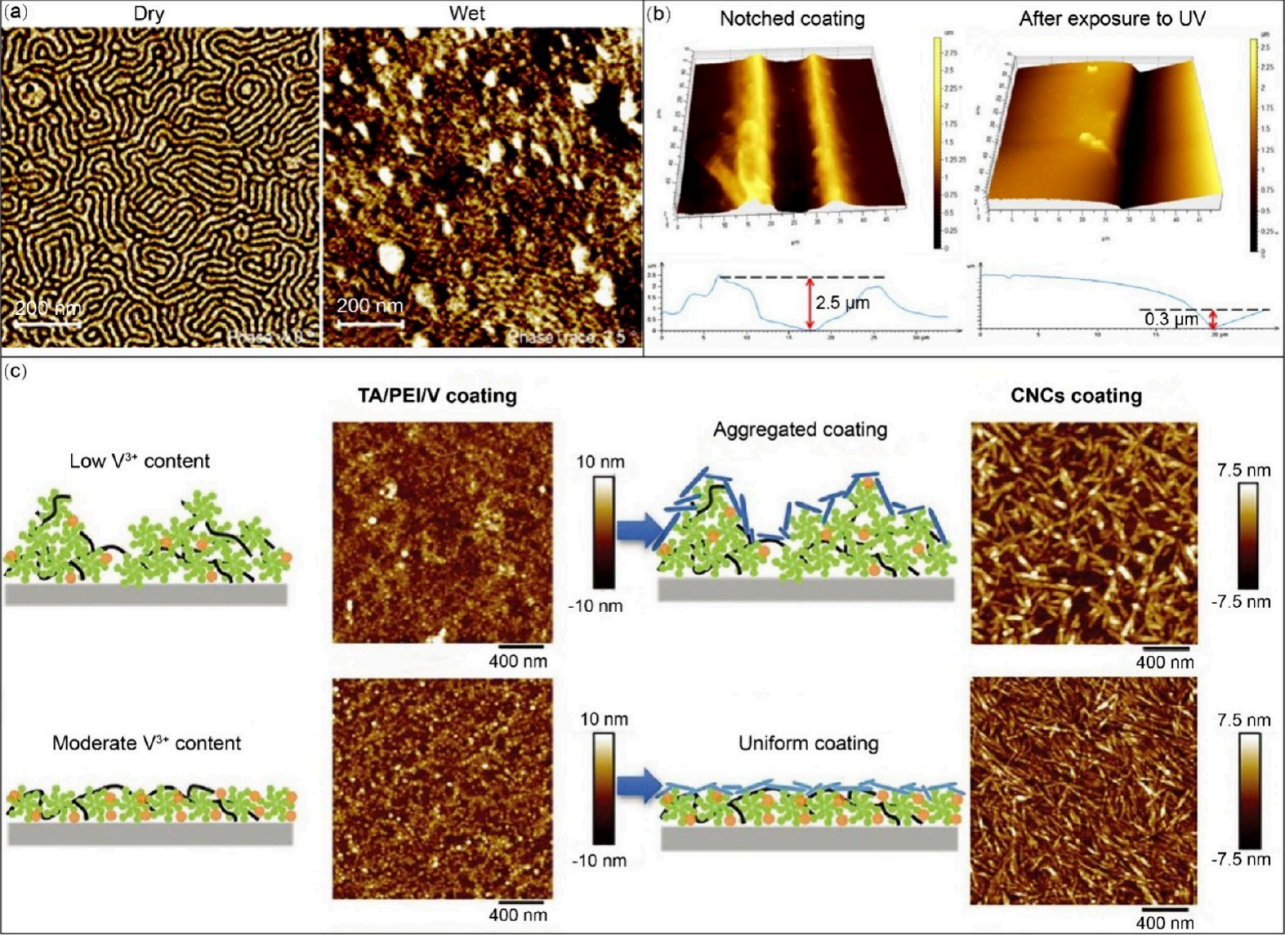
Examples of exploring and optimizing the performance of antifouling coatings using AFM. (a) AFM phase images of S81Sz19_90 recorded after annealing at 120 °C (left) and under artificial seawater after 7 days (right). Reprinted from ref (156). Copyright 2008 American Chemical Society. (b) Self-healing study of notched z-BCP hydrogel having CCL via AFM depth profilometry study after exposure to UV light at *t* = 0 (left) and 120 min (right). Reprinted from ref (160). Copyright 2018 American Chemical Society. (c) Illustrations and topographic AFM images of TA/PEI/V intermediate adhesive layer and corresponding CNCs coatings prepared with different V^3+^ ions content. Reprinted with permission from ref (161). Copyright 2021 Wiley-VCH GmbH.

Callow et al. characterized the surface morphology and roughness of coatings derived from amphiphilic block copolymers with hydrophobic and hydrophilic segments.^156^ The AFM imaging revealed nanostructured domains of discrete polystyrene embedded in an amphiphilic polystyrene matrix, with domain sizes and periodicity dependent on the block copolymer composition (Figure 13(a), left panel). Upon immersion in seawater, AFM imaging revealed a marked transformation toward a more heterogeneous surface architecture accompanied by heightened roughness (Figure 13(a), right panel). The researchers postulated that the molecular and nanoscale ambiguity of the amphiphilic coating diminishes the driving forces for the adsorption of adhesive macromolecules, thereby decreasing the adhesive strength of the organisms.

Walker et al. synthesized nanostructured triblock copolymer films with promising potential as marine antifouling coatings capable of deterring zoospores, barnacles, and tubeworms for a duration of up to one month.^157^ To quantify the adhesive interactions, AFM force spectroscopy was utilized to measure the adhesion forces between bovine serum albumin (BSA)-coated AFM tips and the film surfaces. The significantly reduced adhesion forces observed on the films, in comparison to uncoated surfaces, indicated the coating’s efficacy in resisting protein adsorption. This remarkable property is largely attributed to the steric repulsion generated by the poly(ethylene oxide) (PEO) corona chains that extend in an aqueous environment, overpowering the typically dominant van der Waals attraction between the surface and proteins.

Zhang’s team studied the surface elastic modulus of poly(dimethylsiloxane) (PDMS)-based coatings, leveraging AFM to demonstrate that a low elastic modulus is a critical determinant of the fouling-release potential of silicone elastomers.^158^ Such a low modulus facilitates the generation of a dynamically responsive surface that effectively hampers the tenacious attachment of microorganisms.

Zwitterionic coatings, characterized by their electrostatically induced hydration, exhibit remarkable resistance to nonspecific protein adsorption, bacterial adhesion, and biofilm formation.^159^ This attribute renders zwitterionic coatings especially desirable for antifouling applications. Shaoyi Jiang, a leading expert in the field of zwitterionic materials, and his group extensively investigated the properties and applications of these coatings, contributing significantly to our understanding of their antifouling mechanisms and potential uses. Singha et al. developed a zwitterionic block copolymer hydrogel as an antifouling coating, which exhibited antifouling properties while simultaneously incorporating self-healing functionalities through a synergistic interplay of disulfide metathesis reactions and zwitterionic interactions.^160^ AFM analysis provided quantitative measurements of the notch depth before and after UV exposure, which accurately determined healing efficiency. As shown in Figure 13(b), the coating integrating both disulfide cross-links and zwitterionic segments exhibited the highest healing efficiency (88%), substantiating the complementary effect of these dual healing mechanisms in preserving the coating’s integrity and prolonging its antifouling efficacy.

Zeng et al. addressed the challenge of poor adhesion between antifouling coatings and substrates by tightly anchoring superhydrophilic antifouling cellulose nanocrystals (CNCs) coatings to various substrates via an intermediate adhesive layer composed of tannic acid (TA)/polyethylenimine (PEI)/vanadium(V).^161^ The TA/PEI/V intermediate adhesive layer morphology and the corresponding CNCs coatings were investigated at different V^3+^ concentrations using AFM (Figure 13(c)). The experimental findings revealed that V^3+^ inhibits the reaction between TA and PEI through coordination reactions, thereby controlling the structure of the TA/PEI/V coating. At low V^3+^ concentrations, the TA/PEI/V coating was rough, resulting in an aggregated CNCs coating. Conversely, upon judiciously escalating the V^3+^ concentration, a marked transition to a smoother TA/PEI/V coating was observed, facilitating the dense and uniform deposition of CNCs. This optimized morphology was found to significantly enhance the antifouling properties of the coating, underlining the importance of fine-tuning the V^3+^ content in achieving optimal performance. Moreover, the colloidal probe technique was employed to quantitatively assess the adhesive strength between the TA/PEI/V intermediate layer immobilized on a silica probe and CNCs coating. A strong adhesion force (F/R ≈ 0.62 mN/m) was detected, which could be attributed to the synergistic interplay of multiple noncovalent interactions, including electrostatic interaction, hydrogen bonding, and coordination reaction. These strong interfacial interactions not only ensured the high density and structural stability of the CNCs coating but also contributed to its enhanced adhesion to the substrate. Hence, AFM emerges as an indispensable instrument in understanding the assembly process of coatings and elucidating the fundamental reaction mechanisms.

In summary, the studies highlighted in this section demonstrate the versatility of AFM in probing the complexities of various antifouling coatings. The detailed insights AFM provides into surface morphology, mechanical properties, and interfacial interactions are critical for understanding the mechanisms behind antifouling performance and guiding the design of novel coatings with improved resistance to biofouling. Thus, AFM plays a pivotal role in advancing the field of antifouling coatings by enabling the translation of molecular-level insights into macroscopic properties and performance.

### Application of Bioadhesives

**Figure 14:**
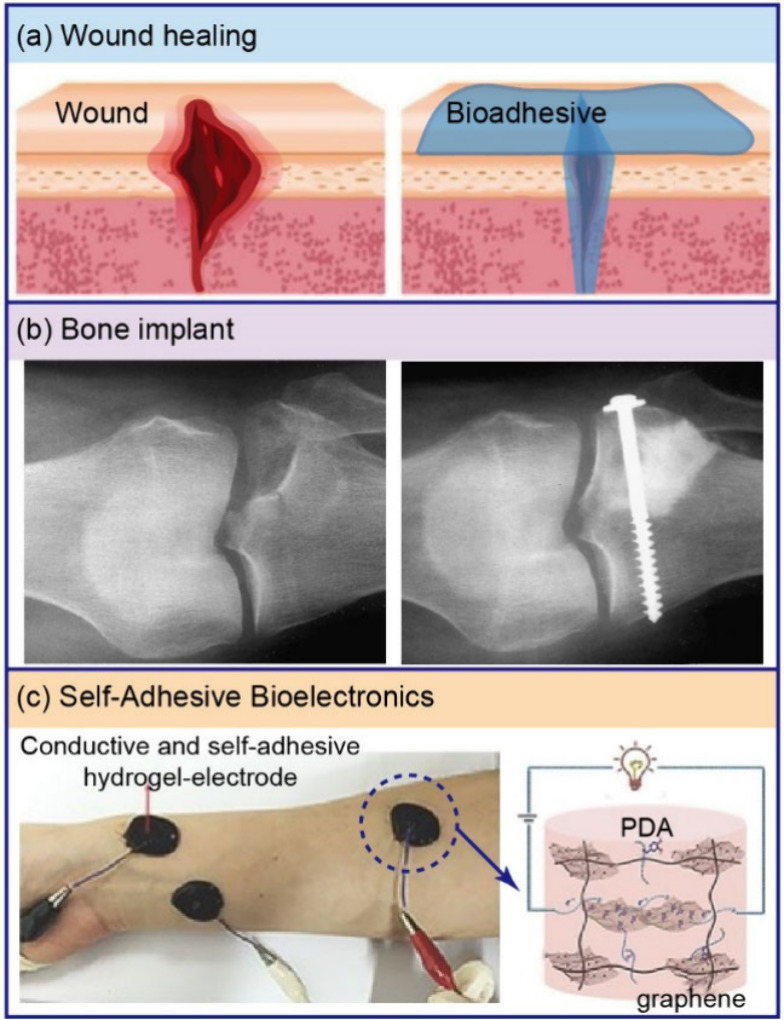
Applications of marine adhesive-inspired materials. (a) Bioadhesives are applied at the outside of wounds for accelerated healing. Reproduced from ref (168) Available under a Creative Commons CC-BY License. Copyright 2021 The Authors. Published by Frontiers Media S.A. (b) A split-depression fracture of the lateral tibial plateau and screw implantation fixation employing bioadhesives. Reproduced with permission from ref (169). Copyright 2001 British Editorial Society of Bone & Joint Surgery. (c) A mussel-inspired conductive, self-adhesive, and self-healable tough hydrogel for use in bioelectronics. Reproduced with permission from ref (170). Copyright 2017 Wiley-VCH Verlag GmbH.

Following research into the bioadhesion mechanisms of marine fouling, more and more bioinspired adhesives are being developed and used in a wide range of applications, including surgical applications, medical implants, and bioelectronics. Inspired by the mussel adhesive proteins, polymers with catechol pendant groups have shown excellent hemostatic properties and wound healing capabilities, making them valuable for surgical applications (Figure 14(a)).^162,163^ The immune response and new tissue formation are important design factors for medical implants. Most recently, implant surfaces have been developed via mussel adhesion-mediated ion coordination and molecular clicking, which can synergistically modulate the osteoimmune microenvironment at the bone-implant interface to promote bone regeneration and facilitate implant success (Figure 14(b)).^164^ Wearable bioelectronic devices face the challenge of achieving adequate and long-term self-adhesion on soft and wet biological tissues. Mussel-inspired hydrogels have emerged as promising materials for self-adhesive bioelectronics, and their latest progress has been summarized (Figure 14(c)).^165,166^ Additionally, reviews on the versatility of catechol-containing polymers in various fields, such as tissue engineering and drug delivery, highlight their significant role in developing advanced biomaterials.^167^

In summary of this section, bioinspired adhesives are revolutionizing the field of adhesion by drawing inspiration from natural mechanisms and translating them into practical applications. Their unique properties and biocompatibility make them invaluable for a wide range of applications, including surgery, medical implants, and bioelectronics. As our understanding of bioadhesion mechanisms continues to grow, the potential for innovative applications of these adhesives is vast.

### Artificial Intelligence Empowering AFM Data Analysis and Material Design

The integration of artificial intelligence (AI) with AFM has opened new avenues for advanced data analysis and material design, significantly enhancing the capabilities of this powerful characterization tool.^171^ This section explores how AI techniques have been applied to AFM data analysis and how they are influencing the future of materials science.

Machine learning (ML) algorithms have proven very effective in automating the laborious and often subjective task of AFM image analysis. Convolutional Neural Networks (CNNs) have been employed to evaluate probe conditions and automatically recondition degraded tips, significantly improving tip quality assessment accuracy.^172^ Additionally, Kocur et al. utilized a ResU-Net architecture to remove artifacts from AFM images, improving topography reconstruction without manual postprocessing.^173^ Müller’s group has leveraged ML techniques to automate the processing of indentation data acquired via AFM.^174^ By correlating subjective human ratings with predefined features extracted from force curves, they accurately classified force curve quality with a precision rate of 87%. Zhou et al. employed Principal Component Analysis (PCA) for dimensionality reduction of single-molecule force spectroscopy data and classification using Support Vector Machines (SVM). This approach enabled precise categorization of force curves into specific, and nonspecific binding, and no interaction scenarios between proteins and ligands.^175^ Furthermore, Ilieva et al. developed an automated clustering method tailored for single-molecule force curves obtained from heterogeneous samples.^176^ These advancements reduce the need for expert intervention and increase the reliability of experimental data. Sokolov et al. developed a novel diagnostic imaging approach for bladder cancer detection by combining AFM with ML.^177^ Moreover, ML was used to analyze AFM force spectroscopy data to predict bacterial viability accurately.^178^

The combination of AFM and AI can be extended to the design of eco-friendly antifouling coatings. Researchers can simulate and predict material behavior under different conditions by leveraging ML’s ability to identify patterns and features within vast data sets. This predictive power facilitates the discovery of new materials with desired properties, optimizing their performance for specific applications. For example, AI can predict the optimal composition and structure of polymer-based coatings with low adhesion and high fouling-release properties.^179^ We note here that integration of AI to marine antifouling studies by AFM has just begun, and besides a few references, there is very little in the literature about this methodological combination. However, this approach not only accelerates the development of new coatings but also ensures that they are environmentally benign, addressing the growing concerns over the ecological impact of traditional antifouling methods.

## Conclusions and Outlook

Natural biological adhesives have attracted considerable attention due to their crucial role in controlling the adhesion of biofouling organisms. The many related, still unsolved questions require new approaches to characterize and elucidate the hitherto unsolved challenges. AFM has emerged as a versatile platform for analyzing these adhesives, providing fundamentally novel insights into foulant-substrate characteristics, including morphology, nanomechanical properties, interactions with interfaces of different properties, as well as insights into processes across the length scales. At the mesoscale, AFM-related methods provide comprehensive and quantitative assessments of the physical, chemical, and surface-active properties of biological adhesives, which play a central role in marine fouling. At the molecular level, AFM-SMFS is a developing area that promises to yield invaluable insights into the adhesive forces of biofouling-related proteins. It can reveal the impact of surface-induced conformational changes on adhesion dynamics, elucidating how specific surface properties, such as chemistry, morphology, as well as surface mechanical properties influence the conformation and adhesive strength of the adhesives.

Developing “smart”, stimuli-responsive coatings that can adapt to changing environmental conditions is an exciting direction for future research. AFM can play a crucial role in characterizing the dynamic behavior of these coatings, including their response to temperature, pH, and mechanical stress. For example, by monitoring real-time changes in surface properties, researchers can optimize the design of coatings that release antifouling agents or change their surface energy in response to fouling pressure. This adaptive behavior can significantly enhance the longevity and effectiveness of antifouling coatings, reducing the need for frequent reapplication and minimizing environmental impact. Future research could focus on developing coatings that respond to multiple environmental cues, such as light, salinity, and biological signals, to further improve their performance. Finally, translating laboratory findings to practical applications remains a critical challenge. To bridge this gap, it is essential to conduct long-term field studies to validate the performance of new antifouling coatings under real-world conditions. AFM can be used to monitor the aging and degradation of coatings over time, providing valuable insights into their durability and efficacy.

Emerging challenges in bioadhesive research necessitate the development of methodologies that can address the complex interplay between bioadhesives and their substrates, especially in marine environments where conditions are highly variable. One significant challenge is understanding the multifaceted nature of marine organism attachment, which involves both biochemical and physical and mechanical interactions. To tackle this challenge, interdisciplinary collaborations between biologists, chemists, engineers, material scientists, and computational experts are indispensable. For instance, integrating AFM with other imaging techniques, like electron microscopy or optical microscopy, can provide complementary information about bioadhesives’ structural and functional aspects. Combining AFM with spectroscopic methods can offer deeper insights into adhesives’ chemical composition and interaction mechanisms. Furthermore, the synergy between AFM and computational modeling can accelerate the discovery of novel adhesives by predicting their performance under diverse conditions.

In conclusion, AFM has become a pivotal platform in comprehending and mitigating marine biofouling, surpassing traditional analytical barriers and promoting multidisciplinary progress. The prospect of employing AI-driven AFM technologies is promising for deciphering the complexities of biofouling attachment in unprecedented detail. This advancement will aid in developing next-generation antifouling strategies and bioadhesives, ensuring they are both effective and environmentally friendly. Future research should focus on the practical application of these advanced techniques and the development of “smart”, stimuli-responsive coatings, paving the way for more sustainable and robust solutions to protect marine infrastructure and ecosystems.

## Vocabulary

Marine biofouling: The unwanted accumulation of microorganisms, plants, and animals on surfaces immersed in seawater, causing structural damage, efficiency loss, and economic impacts.
Adhesive proteins: Adhesive proteins are specialized proteins secreted by marine organisms that enable them to attach to surfaces. Key components like DOPA play a vital role in adhesion.
Single-molecule force spectroscopy (SMFS): SMFS uses AFM to measure the forces required to stretch or pull apart single molecules, providing insights into molecular interactions and conformations.
Sacrificial bonds: Supramolecular and adhesive bonds within biomaterials, like adhesives, that are designed to break preferentially under stress, dissipating energy and increasing material toughness. They allow for reversible adhesion and self-healing properties.
Polymer brushes: Polymer brushes are dense arrays of polymer chains grafted to a surface, with their chemical composition, thickness, density, and flexibility influencing their antifouling performance.

## References

[ref1] Banerjee I.; Pangule R. C.; Kane R. S. Antifouling Coatings: Recent Developments in the Design of Surfaces That Prevent Fouling by Proteins, Bacteria, and Marine Organisms. Adv. Mater. 2011, 23 (6), 690–718. 10.1002/adma.201001215. 20886559

[ref2] Callow J. A.; Callow M. E. Trends in the Development of Environmentally Friendly Fouling-Resistant Marine Coatings. Nat. Commun. 2011, 2, 244 10.1038/ncomms1251. 21427715

[ref3] Genzer J.; Efimenko K. Recent Developments in Superhydrophobic Surfaces and Their Relevance to Marine Fouling: a Review. Biofouling 2006, 22 (5), 339–360. 10.1080/08927010600980223. 17110357

[ref4] Kamino K. Mini-Review: Barnacle Adhesives and Adhesion. Biofouling 2013, 29 (6), 735–749. 10.1080/08927014.2013.800863. 23802872

[ref5] Aldred N.; Clare A. S. The Adhesive Strategies of Cyprids and Development of Barnacle-Resistant Marine Coatings. Biofouling 2008, 24 (5), 351–363. 10.1080/08927010802256117. 18597201

[ref6] Rosenhahn A.; Schilp S.; Kreuzer H. J.; Grunze M. The Role of “Inert” Surface Chemistry in Marine Biofouling Prevention. Phys. Chem. Chem. Phys. 2010, 12 (17), 4275–4286. 10.1039/c001968m. 20407695

[ref7] Callow M. E.; Callow J. A.; Pickett-Heaps J. D.; Wetherbee R. Primary Adhesion of *Enteromorpha* (Chlorophyta, Ulvales) Propagules: Quantitative Settlement Studies and Video Microscopy. J. Phycol. 1997, 33 (6), 938–947. 10.1111/j.0022-3646.1997.00938.x.

[ref8] Roberts D.; Rittschof D.; Holm E.; Schmidt A. Factors Influencing Initial Larval Settlement: Temporal, Spatial and Surface Molecular Components. J. Exp. Mar. Biol. Ecol. 1991, 150 (2), 203–221. 10.1016/0022-0981(91)90068-8.

[ref9] Schultz M. P. Effects of Coating Roughness and Biofouling on Ship Resistance and Powering. Biofouling 2007, 23 (5), 331–341. 10.1080/08927010701461974. 17852068

[ref10] Corbett J. J.; Koehler H. W. Updated Emissions from Ocean Shipping. J. Geophys. Res.-Atmos. 2003, 108 (D20), 4650 10.1029/2003JD003751.

[ref11] Lejars M.; Margaillan A.; Bressy C. Fouling Release Coatings: a Nontoxic Alternative to Biocidal Antifouling Coatings. Chem. Rev. 2012, 112 (8), 4347–4390. 10.1021/cr200350v. 22578131

[ref12] Bannister J.; Sievers M.; Bush F.; Bloecher N. Biofouling in Marine Aquaculture: a Review of Recent Research and Developments. Biofouling 2019, 35 (6), 631–648. 10.1080/08927014.2019.1640214. 31339358

[ref13] Leonardi A. K.; Ober C. K. Polymer-Based Marine Antifouling and Fouling Release Surfaces: Strategies for Synthesis and Modification. Annu. Rev. Chem. Biomol. Engineer. 2019, 10, 241–264. 10.1146/annurev-chembioeng-060718-030401. 31173523

[ref14] Detty M. R.; Ciriminna R.; Bright F. V.; Pagliaro M. Environmentally Benign Sol-Gel Antifouling and Foul-Releasing Coatings. Acc. Chem. Res. 2014, 47 (2), 678–687. 10.1021/ar400240n. 24397288

[ref15] Ytreberg E.; Karlsson J.; Eklund B. Comparison of Toxicity and Release Rates of Cu and Zn from Anti-Fouling Paints Leached in Natural and Artificial Brackish Seawater. Sci. Total Environ. 2010, 408 (12), 2459–2466. 10.1016/j.scitotenv.2010.02.036. 20347476

[ref16] Kirschner C. M.; Brennan A. B. Bio-Inspired Antifouling Strategies. Annu. Rev. Mater. Res. 2012, 42, 211–229. 10.1146/annurev-matsci-070511-155012.

[ref17] Magin C. M.; Cooper S. P.; Brennan A. B. Non-Toxic Antifouling Strategies. Mater. Today 2010, 13 (4), 36–44. 10.1016/S1369-7021(10)70058-4.

[ref18] Zhao K.; Li M.; Zhang P.; Cui J. Sticktight-Inspired Pegylation for Low-Fouling Coatings. Chem. Commun. 2022, 58 (99), 13735–13738. 10.1039/D2CC04938D. 36415979

[ref19] Nir S.; Reches M. Bio-Inspired Antifouling Approaches: the Quest Towards Non-Toxic and Non-Biocidal Materials. Curr. Opin. Biotechnol. 2016, 39, 48–55. 10.1016/j.copbio.2015.12.012. 26773304

[ref20] Xie Q.; Pan J.; Ma C.; Zhang G. Dynamic Surface Antifouling: Mechanism and Systems. Soft Matter 2019, 15 (6), 1087–1107. 10.1039/C8SM01853G. 30444519

[ref21] Howell C.; Grinthal A.; Sunny S.; Aizenberg M.; Aizenberg J. Designing Liquid-Infused Surfaces for Medical Applications: a Review. Adv. Mater. 2018, 30 (50), 1802724 10.1002/adma.201802724. 30151909

[ref22] Yebra D. M.; Kiil S.; Dam-Johansen K. Antifouling Technology—Past, Present and Future Steps Towards Efficient and Environmentally Friendly Antifouling Coatings. Prog. Org. Coat. 2004, 50 (2), 75–104. 10.1016/j.porgcoat.2003.06.001.

[ref23] Maréchal J.-P.; Hellio C. Challenges for the Development of New Non-Toxic Antifouling Solutions. Int. J. Mol. Sci. 2009, 10 (11), 4623–4637. 10.3390/ijms10114623. 20087457 PMC2808003

[ref24] Hennebert E.; Maldonado B.; Ladurner P.; Flammang P.; Santos R. Experimental Strategies for the Identification and Characterization of Adhesive Proteins in Animals: a Review. Interface Focus 2015, 5 (1), 20140064 10.1098/rsfs.2014.0064. 25657842 PMC4275877

[ref25] Petrone L.; Kumar A.; Sutanto C. N.; Patil N. J.; Kannan S.; Palaniappan A.; Amini S.; Zappone B.; Verma C.; Miserez A. Mussel Adhesion Is Dictated by Time-Regulated Secretion and Molecular Conformation of Mussel Adhesive Proteins. Nat. Commun. 2015, 6 (1), 1–12. 10.1038/ncomms9737. PMC4640085 26508080

[ref26] Hinterdorfer P.; Dufrêne Y. F. Detection and Localization of Single Molecular Recognition Events Using Atomic Force Microscopy. Nat. Methods 2006, 3 (5), 347–355. 10.1038/nmeth871. 16628204

[ref27] Dufrêne Y. F.; Ando T.; Garcia R.; Alsteens D.; Martinez-Martin D.; Engel A.; Gerber C.; Müller D. J. Imaging Modes of Atomic Force Microscopy for Application in Molecular and Cell Biology. Nat. Nanotechnol. 2017, 12 (4), 295–307. 10.1038/nnano.2017.45. 28383040

[ref28] Alsteens D.; Gaub H. E.; Newton R.; Pfreundschuh M.; Gerber C.; Müller D. J. Atomic Force Microscopy-Based Characterization and Design of Biointerfaces. Nat. Rev. Mater. 2017, 2 (5), 1–16. 10.1038/natrevmats.2017.8.

[ref29] Dufrêne Y. F.; Martínez-Martín D.; Medalsy I.; Alsteens D.; Müller D. J. Multiparametric Imaging of Biological Systems by Force-Distance Curve-Based AFM. Nat. Methods 2013, 10 (9), 847–854. 10.1038/nmeth.2602. 23985731

[ref30] Garcia R. Nanomechanical Mapping of Soft Materials with the Atomic Force Microscope: Methods, Theory and Applications. Chem. Soc. Rev. 2020, 49 (16), 5850–5884. 10.1039/D0CS00318B. 32662499

[ref31] Petrosyan R.; Narayan A.; Woodside M. T. Single-Molecule Force Spectroscopy of Protein Folding. J. Mol. Biol. 2021, 433 (20), 167207 10.1016/j.jmb.2021.167207. 34418422

[ref32] Jacobson D. R.; Perkins T. T. Free-Energy Changes of Bacteriorhodopsin Point Mutants Measured by Single-Molecule Force Spectroscopy. Proc. Natl. Acad. Sci. U. S. A. 2021, 118 (13), e2020083118 10.1073/pnas.2020083118. 33753487 PMC8020790

[ref33] Lipke P. N.; Rauceo J. M.; Viljoen A. Cell-Cell Mating Interactions: Overview and Potential of Single-Cell Force Spectroscopy. Int. J. Mol. Sci. 2022, 23 (3), 1110 10.3390/ijms23031110. 35163034 PMC8835621

[ref34] Herman-Bausier P.; Labate C.; Towell A. M.; Derclaye S.; Geoghegan J. A.; Dufrêne Y. F. *Staphylococcus aureus* Clumping Factor a Is a Force-Sensitive Molecular Switch That Activates Bacterial Adhesion. Proc. Natl. Acad. Sci. U. S. A. 2018, 115 (21), 5564–5569. 10.1073/pnas.1718104115. 29735708 PMC6003445

[ref35] Viljoen A.; Mathelié-Guinlet M.; Ray A.; Strohmeyer N.; Oh Y. J.; Hinterdorfer P.; Müller D. J.; Alsteens D.; Dufrêne Y. F. Force Spectroscopy of Single Cells Using Atomic Force Microscopy. Nat. Rev. Methods Primers 2021, 1 (1), 1–24. 10.1038/s43586-021-00062-x.

[ref36] Neuman K. C.; Nagy A. Single-Molecule Force Spectroscopy: Optical Tweezers, Magnetic Tweezers and Atomic Force Microscopy. Nat. Methods 2008, 5 (6), 491–505. 10.1038/nmeth.1218. 18511917 PMC3397402

[ref37] Müller D. J.; Dufrêne Y. F. Atomic Force Microscopy as a Multifunctional Molecular Toolbox in Nanobiotechnology. Nat. Nanotechnol. 2008, 3 (5), 261–269. 10.1038/nnano.2008.100. 18654521

[ref38] Giessibl F. J. Advances in Atomic Force Microscopy. Rev. Mod. Phys. 2003, 75 (3), 949–983. 10.1103/RevModPhys.75.949.

[ref39] Krieg M.; Fläschner G.; Alsteens D.; Gaub B. M.; Roos W. H.; Wuite G. J. L.; Gaub H. E.; Gerber C.; Dufrêne Y. F.; Müller D. J. Atomic Force Microscopy-Based Mechanobiology. Nat. Rev. Phys. 2019, 1 (1), 41–57. 10.1038/s42254-018-0001-7.

[ref40] Voigtländer B. Atomic Force Microscopy; Springer: Switzerland, 2019; pp 137–259.

[ref41] Dumitru A. C.; Mohammed D.; Maja M.; Yang J.; Verstraeten S.; Del Campo A.; Mingeot-Leclercq M. P.; Tyteca D.; Alsteens D. Label-Free Imaging of Cholesterol Assemblies Reveals Hidden Nanomechanics of Breast Cancer Cells. Adv. Sci. 2020, 7 (22), 2002643 10.1002/advs.202002643. PMC7675049 33240781

[ref42] Hadfield M. G. Biofilms and Marine Invertebrate Larvae: What Bacteria Produce That Larvae Use to Choose Settlement Sites. Annu. Rev. Mar. Sci. 2011, 3, 453–470. 10.1146/annurev-marine-120709-142753. 21329213

[ref43] Flemming H. C.; Wingender J.; Szewzyk U.; Steinberg P.; Rice S. A.; Kjelleberg S. Biofilms: an Emergent Form of Bacterial Life. Nat. Rev. Microbiol. 2016, 14 (9), 563–575. 10.1038/nrmicro.2016.94. 27510863

[ref44] Flemming H. C.; Wingender J. The Biofilm Matrix. Nat. Rev. Microbiol. 2010, 8 (9), 623–633. 10.1038/nrmicro2415. 20676145

[ref45] Hori K.; Matsumoto S. Bacterial Adhesion: From Mechanism to Control. Biochem. Eng. J. 2010, 48 (3), 424–434. 10.1016/j.bej.2009.11.014.

[ref46] Bhaskar P.; Grossart H.-P.; Bhosle N.; Simon M. Production of Macroaggregates from Dissolved Exopolymeric Substances (EPS) of Bacterial and Diatom Origin. FEMS Microbiol. Ecol. 2005, 53 (2), 255–264. 10.1016/j.femsec.2004.12.013. 16329945

[ref47] Molino P. J.; Wetherbee R. The Biology of Biofouling Diatoms and Their Role in the Development of Microbial Slimes. Biofouling 2008, 24 (5), 365–379. 10.1080/08927010802254583. 18604655

[ref48] Thompson S. E.; Coates J. C. Surface Sensing and Stress-Signalling in *Ulva* and Fouling Diatoms-Potential Targets for Antifouling: a Review. Biofouling 2017, 33 (5), 410–432. 10.1080/08927014.2017.1319473. 28508711

[ref49] Callow M. E.; Callow J. Substratum Location and Zoospore Behaviour in the Fouling Alga *Enteromorpha*. Biofouling 2000, 15 (1–3), 49–56. 10.1080/08927010009386297. 22115291

[ref50] Heydt M.; Rosenhahn A.; Grunze M.; Pettitt M.; Callow M.; Callow J. Digital in-Line Holography as a Three-Dimensional Tool to Study Motile Marine Organisms During Their Exploration of Surfaces. J. Adhes. 2007, 83 (5), 417–430. 10.1080/00218460701377388.

[ref51] Liang C.; Ye Z.; Xue B.; Zeng L.; Wu W.; Zhong C.; Cao Y.; Hu B.; Messersmith P. B. Self-Assembled Nanofibers for Strong Underwater Adhesion: the Trick of Barnacles. ACS Appl. Mater. Interfaces 2018, 10 (30), 25017–25025. 10.1021/acsami.8b04752. 29990429

[ref52] Phang I. Y.; Aldred N.; Ling X. Y.; Huskens J.; Clare A. S.; Vancso G. J. Atomic Force Microscopy of the Morphology and Mechanical Behaviour of Barnacle Cyprid Footprint Proteins at the Nanoscale. J. R. Soc., Interface 2010, 7 (43), 285–296. 10.1098/rsif.2009.0127. 19570797 PMC2842607

[ref53] Phang I. Y.; Aldred N.; Clare A. S.; Callow J. A.; Vancso G. J. An *in Situ* Study of the Nanomechanical Properties of Barnacle (*Balanus Amphitrite*) Cyprid Cement Using Atomic Force Microscopy (AFM). Biofouling 2006, 22 (4), 245–250. 10.1080/08927010600857686. 17290868

[ref54] Wiegemann M. Adhesion in Blue Mussels (*Mytilus edulis*) and Barnacles (Genus *Balanus*): Mechanisms and Technical Applications. Aquat. Sci. 2005, 67 (2), 166–176. 10.1007/s00027-005-0758-5.

[ref55] Coyne K. J.; Qin X.-X.; Waite J. H. Extensible Collagen in Mussel Byssus: a Natural Block Copolymer. Science 1997, 277 (5333), 1830–1832. 10.1126/science.277.5333.1830. 9295275

[ref56] Lee B. P.; Messersmith P. B.; Israelachvili J. N.; Waite J. H. Mussel-Inspired Adhesives and Coatings. Annu. Rev. Mater. Res. 2011, 41, 99–132. 10.1146/annurev-matsci-062910-100429. 22058660 PMC3207216

[ref57] Wei W.; Yu J.; Broomell C.; Israelachvili J. N.; Waite J. H. Hydrophobic Enhancement of Dopa-Mediated Adhesion in a Mussel Foot Protein. J. Am. Chem. Soc. 2013, 135 (1), 377–383. 10.1021/ja309590f. 23214725 PMC3587158

[ref58] Waite J. H. Mussel Adhesion-Essential Footwork. J. Exp. Biol. 2017, 220 (4), 517–530. 10.1242/jeb.134056. 28202646 PMC5312731

[ref59] He Y.; Sun C.; Jiang F.; Yang B.; Li J.; Zhong C.; Zheng L.; Ding H. Lipids as Integral Components in Mussel Adhesion. Soft Matter 2018, 14 (35), 7145–7154. 10.1039/C8SM00509E. 29978875

[ref60] Narayanan A.; Dhinojwala A.; Joy A. Design Principles for Creating Synthetic Underwater Adhesives. Chem. Soc. Rev. 2021, 50 (23), 13321–13345. 10.1039/D1CS00316J. 34751690

[ref61] Dufrêne Y. F.; Boonaert C. J.; Gerin P. A.; Asther M.; Rouxhet P. G. Direct Probing of the Surface Ultrastructure and Molecular Interactions of Dormant and Germinating Spores of *Phanerochaete Chrysosporium*. J. Bacteriol. 1999, 181 (17), 5350–5354. 10.1128/JB.181.17.5350-5354.1999. 10464206 PMC94041

[ref62] Dickinson G. H.; Vega I. E.; Wahl K. J.; Orihuela B.; Beyley V.; Rodriguez E. N.; Everett R. K.; Bonaventura J.; Rittschof D. Barnacle Cement: a Polymerization Model Based on Evolutionary Concepts. J. Exp. Biol. 2009, 212 (21), 3499–3510. 10.1242/jeb.029884. 19837892 PMC2762877

[ref63] Sullan R. M. A.; Gunari N.; Tanur A. E.; Chan Y.; Dickinson G. H.; Orihuela B.; Rittschof D.; Walker G. C. Nanoscale Structures and Mechanics of Barnacle Cement. Biofouling 2009, 25 (3), 263–275. 10.1080/08927010802688095. 19180351

[ref64] Berglin M.; Gatenholm P. The Barnacle Adhesive Plaque: Morphological and Chemical Differences as a Response to Substrate Properties. Colloid Surf. B-Biointerfaces 2003, 28 (2–3), 107–117. 10.1016/S0927-7765(02)00149-2.

[ref65] Guo S.; Puniredd S. R.; Jańczewski D.; Lee S. S. C.; Teo S. L. M.; He T.; Zhu X.; Vancso G. J. Barnacle Larvae Exploring Surfaces with Variable Hydrophilicity: Influence of Morphology and Adhesion of “Footprint” Proteins by AFM. ACS Appl. Mater. Interfaces 2014, 6 (16), 13667–13676. 10.1021/am503147m. 25055115

[ref66] Chopinet L.; Formosa C.; Rols M.; Duval R.; Dague E. Imaging Living Cells Surface and Quantifying Its Properties at High Resolution Using AFM in QI Mode. Micron 2013, 48, 26–33. 10.1016/j.micron.2013.02.003. 23522742

[ref67] Phang I. Y.; Aldred N.; Clare A. S.; Vancso G. J. Towards a Nanomechanical Basis for Temporary Adhesion in Barnacle Cyprids (*Semibalanus balanoides*). J. R. Soc., Interface 2008, 5 (21), 397–402. 10.1098/rsif.2007.1209. 17971318 PMC2607389

[ref68] Phang I. Y.; Aldred N.; Ling X. Y.; Tomczak N.; Huskens J.; Clare A. S.; Vancso G. J. Chemistry-Specific Interfacial Forces between Barnacle (*Semibalanus balanoides*) Cyprid Footprint Proteins and Chemically Functionalised AFM Tips. J. Adhes. 2009, 85 (9), 616–630. 10.1080/00218460902996952.

[ref69] Phang I. Y.; Chaw K. C.; Choo S. S. H.; Kang R. K. C.; Lee S. S. C.; Birch W. R.; Teo S. L. M.; Vancso G. J. Marine Biofouling Field Tests, Settlement Assay and Footprint Micromorphology of Cyprid Larvae of *Balanus Amphitrite* on Model Surfaces. Biofouling 2009, 25 (2), 139–147. 10.1080/08927010802592925. 19031305

[ref70] Malfatti F.; Samo T. J.; Azam F. High-Resolution Imaging of Pelagic Bacteria by Atomic Force Microscopy and Implications for Carbon Cycling. ISME J. 2010, 4 (3), 427–439. 10.1038/ismej.2009.116. 19940866

[ref71] Callow J.; Crawford S.; Higgins M.; Mulvaney P.; Wetherbee R. The Application of Atomic Force Microscopy to Topographical Studies and Force Measurements on the Secreted Adhesive of the Green Alga *Enteromorpha*. Planta 2000, 211 (5), 641–647. 10.1007/s004250000337. 11089676

[ref72] Pfreundschuh M.; Martinez-Martin D.; Mulvihill E.; Wegmann S.; Muller D. J. Multiparametric High-Resolution Imaging of Native Proteins by Force-Distance Curve-Based AFM. Nat. Protoc. 2014, 9 (5), 1113–1130. 10.1038/nprot.2014.070. 24743419

[ref73] George M. N.; Carrington E. Environmental Post-Processing Increases the Adhesion Strength of Mussel Byssus Adhesive. Biofouling 2018, 34 (4), 388–397. 10.1080/08927014.2018.1453927. 29637795

[ref74] Higgins M. J.; Crawford S. A.; Mulvaney P.; Wetherbee R. Characterization of the Adhesive Mucilages Secreted by Live Diatom Cells Using Atomic Force Microscopy. Protist 2002, 153 (1), 25–38. 10.1078/1434-4610-00080. 12022272

[ref75] Sun Y.; Guo S.; Walker G. C.; Kavanagh C. J.; Swain G. W. Surface Elastic Modulus of Barnacle Adhesive and Release Characteristics from Silicone Surfaces. Biofouling 2004, 20 (6), 279–289. 10.1080/08927010400026383. 15804712

[ref76] Walker G. C.; Sun Y.; Guo S.; Finlay J. A.; Callow M. E.; Callow J. A. Surface Mechanical Properties of the Spore Adhesive of the Green Alga *Ulva*. J. Adhes. 2005, 81 (10–11), 1101–1118. 10.1080/00218460500310846.

[ref77] Kumar U.; Vivekanand K.; Poddar P. Real-Time Nanomechanical and Topographical Mapping on Live Bacterial Cells—*Brevibacterium casei* under Stress Due to Their Exposure to Co^2+^ Ions During Microbial Synthesis of Co_3_o_4_ Nanoparticles. J. Phys. Chem. B 2009, 113 (22), 7927–7933. 10.1021/jp902698n. 19438181

[ref78] Abu-Lail N. I.; Camesano T. A. Role of Ionic Strength on the Relationship of Biopolymer Conformation, DLVO Contributions, and Steric Interactions to Bioadhesion of *Pseudomonas putida* KT2442. Biomacromolecules 2003, 4 (4), 1000–1012. 10.1021/bm034055f. 12857085

[ref79] Benaglia S.; Gisbert V. G.; Perrino A. P.; Amo C. A.; Garcia R. Fast and High-Resolution Mapping of Elastic Properties of Biomolecules and Polymers with Bimodal AFM. Nat. Protoc. 2018, 13 (12), 2890–2907. 10.1038/s41596-018-0070-1. 30446750

[ref80] Killgore J. P.; Yablon D.; Tsou A.; Gannepalli A.; Yuya P.; Turner J.; Proksch R.; Hurley D. Viscoelastic Property Mapping with Contact Resonance Force Microscopy. Langmuir 2011, 27 (23), 13983–13987. 10.1021/la203434w. 22054300

[ref81] Kocun M.; Labuda A.; Meinhold W.; Revenko I.; Proksch R. Fast, High Resolution, and Wide Modulus Range Nanomechanical Mapping with Bimodal Tapping Mode. ACS Nano 2017, 11 (10), 10097–10105. 10.1021/acsnano.7b04530. 28953363

[ref82] Galluzzi M.; Tang G.; Biswas C. S.; Zhao J.; Chen S.; Stadler F. J. Atomic Force Microscopy Methodology and Afmech Suite Software for Nanomechanics on Heterogeneous Soft Materials. Nat. Commun. 2018, 9, 3584 10.1038/s41467-018-05902-1. 30181577 PMC6123404

[ref83] Erath J.; Schmidt S.; Fery A. Characterization of Adhesion Phenomena and Contact of Surfaces by Soft Colloidal Probe AFM. Soft Matter 2010, 6 (7), 1432–1437. 10.1039/b923540j.

[ref84] Ducker W. A.; Senden T. J.; Pashley R. M. Direct Measurement of Colloidal Forces Using an Atomic Force Microscope. Nature 1991, 353 (6341), 239–241. 10.1038/353239a0.

[ref85] Indrieri M.; Podestà A.; Bongiorno G.; Marchesi D.; Milani P. Adhesive-Free Colloidal Probes for Nanoscale Force Measurements: Production and Characterization. Rev. Sci. Instrum. 2011, 82 (2), 023708 10.1063/1.3553499. 21361602

[ref86] Raiteri R.; Preuss M.; Grattarola M.; Butt H.-J. Preliminary Results on the Electrostatic Double-Layer Force between Two Surfaces with High Surface Potentials. Colloid Surf. A-Physicochem. Eng. Asp. 1998, 136 (1–2), 191–197. 10.1016/S0927-7757(97)00339-7.

[ref87] Mittelviefhaus M.; Müller D. B.; Zambelli T.; Vorholt J. A. A Modular Atomic Force Microscopy Approach Reveals a Large Range of Hydrophobic Adhesion Forces among Bacterial Members of the Leaf Microbiota. ISME J. 2019, 13 (7), 1878–1882. 10.1038/s41396-019-0404-1. 30894689 PMC6591122

[ref88] Eskhan A.; Johnson D. Microscale Characterization of Abiotic Surfaces and Prediction of Their Biofouling/Anti-Biofouling Potential Using the AFM Colloidal Probe Technique. Adv. Colloid Interface Sci. 2022, 310, 102796 10.1016/j.cis.2022.102796. 36283341

[ref89] Puricelli L.; Galluzzi M.; Schulte C.; Podestà A.; Milani P. Nanomechanical and Topographical Imaging of Living Cells by Atomic Force Microscopy with Colloidal Probes. Rev. Sci. Instrum. 2015, 86 (3), 033705 10.1063/1.4915896. 25832236

[ref90] Zhu X.; Guo S.; He T.; Jiang S.; Jańczewski D.; Vancso G. J. Engineered, Robust Polyelectrolyte Multilayers by Precise Control of Surface Potential for Designer Protein, Cell, and Bacteria Adsorption. Langmuir 2016, 32 (5), 1338–1346. 10.1021/acs.langmuir.5b04118. 26756285

[ref91] Guo S.; Quintana R.; Cirelli M.; Toa Z. S. D.; Arjunan Vasantha V.; Kooij E. S.; Jańczewski D.; Vancso G. J. Brush Swelling and Attachment Strength of Barnacle Adhesion Protein on Zwitterionic Polymer Films as a Function of Macromolecular Structure. Langmuir 2019, 35 (24), 8085–8094. 10.1021/acs.langmuir.9b00918. 31099575 PMC6587155

[ref92] Guo S.; Zhu X.; Jańczewski D.; Lee S. S. C.; He T.; Teo S. L. M.; Vancso G. J. Measuring Protein Isoelectric Points by AFM-Based Force Spectroscopy Using Trace Amounts of Sample. Nat. Nanotechnol. 2016, 11 (9), 817–823. 10.1038/nnano.2016.118. 27454881

[ref93] Bremmell K. E.; Kingshott P.; Ademovic Z.; Winther-Jensen B.; Griesser H. J. Colloid Probe AFM Investigation of Interactions between Fibrinogen and Peg-Like Plasma Polymer Surfaces. Langmuir 2006, 22 (1), 313–318. 10.1021/la052143a. 16378437

[ref94] Lower S. K.; Tadanier C. J.; Hochella M. F. Jr Measuring Interfacial and Adhesion Forces between Bacteria and Mineral Surfaces with Biological Force Microscopy. Geochim. Cosmochim. Acta 2000, 64 (18), 3133–3139. 10.1016/S0016-7037(00)00430-0.

[ref95] Kang T.; Banquy X.; Heo J.; Lim C.; Lynd N. A.; Lundberg P.; Oh D. X.; Lee H.-K.; Hong Y.-K.; Hwang D. S.; et al. Mussel-Inspired Anchoring of Polymer Loops That Provide Superior Surface Lubrication and Antifouling Properties. ACS Nano 2016, 10 (1), 930–937. 10.1021/acsnano.5b06066. 26695175 PMC4932843

[ref96] Danner E. W.; Kan Y.; Hammer M. U.; Israelachvili J. N.; Waite J. H. Adhesion of Mussel Foot Protein Mefp-5 to Mica: an Underwater Superglue. Biochemistry 2012, 51 (33), 6511–6518. 10.1021/bi3002538. 22873939 PMC3428132

[ref97] Israelachvili J.; Min Y.; Akbulut M.; Alig A.; Carver G.; Greene W.; Kristiansen K.; Meyer E.; Pesika N.; Rosenberg K.; Zeng H. Recent Advances in the Surface Forces Apparatus (SFA) Technique. Rep. Prog. Phys. 2010, 73 (3), 036601 10.1088/0034-4885/73/3/036601.

[ref98] Geng H.; Zhang P.; Peng Q.; Cui J.; Hao J.; Zeng H. Principles of Cation- Π Interactions for Engineering Mussel-Inspired Functional Materials. Acc. Chem. Res. 2022, 55 (8), 1171–1182. 10.1021/acs.accounts.2c00068. 35344662

[ref99] Li M.; Gao Z.; Cui J. Modulation of Colloidal Particle Stiffness for the Exploration of Bio-Nano Interactions. Langmuir 2022, 38 (22), 6780–6785. 10.1021/acs.langmuir.2c01117. 35617605

[ref100] McGuiggan P. M.; Zhang J.; Hsu S. M. Comparison of Friction Measurements Using the Atomic Force Microscope and the Surface Forces Apparatus: the Issue of Scale. Tribol. Lett. 2001, 10 (4), 217–223. 10.1023/A:1016692704748.

[ref101] Li Y.; Cheng J.; Delparastan P.; Wang H.; Sigg S. J.; DeFrates K. G.; Cao Y.; Messersmith P. B. Molecular Design Principles of Lysine-DOPA Wet Adhesion. Nat. Commun. 2020, 11 (1), 1–8. 10.1038/s41467-020-17597-4. 32753588 PMC7403305

[ref102] Vogel V.; Thomas W. E.; Craig D. W.; Krammer A.; Baneyx G. Structural Insights into the Mechanical Regulation of Molecular Recognition Sites. Trends Biotechnol. 2001, 19 (10), 416–423. 10.1016/S0167-7799(01)01737-1. 11587768

[ref103] Meadows P. Y.; Bemis J. E.; Walker G. C. Single-Molecule Force Spectroscopy of Isolated and Aggregated Fibronectin Proteins on Negatively Charged Surfaces in Aqueous Liquids. Langmuir 2003, 19 (23), 9566–9572. 10.1021/la035217w.

[ref104] Fuhrmann A.; Schoening J. C.; Anselmetti D.; Staiger D.; Ros R. Quantitative Analysis of Single-Molecule RNA-Protein Interaction. Biophys. J. 2009, 96 (12), 5030–5039. 10.1016/j.bpj.2009.03.022. 19527663 PMC2712025

[ref105] Liu Y.; Vancso G. J. Polymer Single Chain Imaging, Molecular Forces, and Nanoscale Processes by Atomic Force Microscopy: The Ultimate Proof of the Macromolecular Hypothesis. Prog. Polym. Sci. 2020, 104, 101232 10.1016/j.progpolymsci.2020.101232.

[ref106] Das P.; Reches M. Insights into the Interactions of Amino Acids and Peptides with Inorganic Materials Using Single Molecule Force Spectroscopy. Pept. Sci. 2015, 104 (5), 480–494. 10.1002/bip.22655. 25851866

[ref107] Samorì P. Scanning Probe Microscopies Beyond Imaging. J. Mater. Chem. 2004, 14 (9), 1353–1366. 10.1039/B314626J.

[ref108] Fernandez J. M.; Li H. B. Force-Clamp Spectroscopy Monitors the Folding Trajectory of a Single Protein. Science 2004, 303 (5664), 1674–1678. 10.1126/science.1092497. 15017000

[ref109] Florin E.-L.; Moy V. T.; Gaub H. E. Adhesion Forces between Individual Ligand-Receptor Pairs. Science 1994, 264 (5157), 415–417. 10.1126/science.8153628. 8153628

[ref110] Hugel T.; Seitz M. The Study of Molecular Interactions by AFM Force Spectroscopy. Macromol. Rapid Commun. 2001, 22 (13), 989–1016. 10.1002/1521-3927(20010901)22:13<989::AID-MARC989>3.0.CO;2-D.

[ref111] Duwez A.-S.; Cuenot S.; Jérôme C.; Gabriel S.; Jérôme R.; Rapino S.; Zerbetto F. Mechanochemistry: Targeted Delivery of Single Molecules. Nat. Nanotechnol. 2006, 1 (2), 122–125. 10.1038/nnano.2006.92. 18654163

[ref112] Barattin R.; Voyer N. Chemical Modifications of Atomic Force Microscopy Tips; Humana Press: Totowa, NJ, 2011; pp 457–483. 10.1007/978-1-61779-105-5_28 21660744

[ref113] Vericat C.; Vela M.; Benitez G.; Carro P.; Salvarezza R. Self-Assembled Monolayers of Thiols and Dithiols on Gold: New Challenges for a Well-Known System. Chem. Soc. Rev. 2010, 39 (5), 1805–1834. 10.1039/b907301a. 20419220

[ref114] Wenzler L.; Moyes G.; Olson L.; Harris J.; Beebe T. Single-Molecule Bond-Rupture Force Analysis of Interactions between AFM Tips and Substrates Modified with Organosilanes. Anal. Chem. 1997, 69 (14), 2855–2861. 10.1021/ac961065g.

[ref115] Aissaoui N.; Bergaoui L.; Landoulsi J.; Lambert J.-F.; Boujday S. Silane Layers on Silicon Surfaces: Mechanism of Interaction, Stability, and Influence on Protein Adsorption. Langmuir 2012, 28 (1), 656–665. 10.1021/la2036778. 22107153

[ref116] Wildling L.; Unterauer B.; Zhu R.; Rupprecht A.; Haselgrübler T.; Rankl C.; Ebner A.; Vater D.; Pollheimer P.; Pohl E. E.; et al. Linking of Sensor Molecules with Amino Groups to Amino-Functionalized AFM Tips. Bioconjugate Chem. 2011, 22 (6), 1239–1248. 10.1021/bc200099t. PMC3115690 21542606

[ref117] Yam C.-M.; Xiao Z.; Gu J.; Boutet S.; Cai C. Modification of Silicon AFM Cantilever Tips with an Oligo (Ethylene Glycol) Derivative for Resisting Proteins and Maintaining a Small Tip Size for High-Resolution Imaging. J. Am. Chem. Soc. 2003, 125 (25), 7498–7499. 10.1021/ja0350435. 12812473

[ref118] Barattin R.; Voyer N. Chemical Modifications of AFM Tips for the Study of Molecular Recognition Events. Chem. Commun. 2008, 13, 1513–1532. 10.1039/b614328h. 18354789

[ref119] Janshoff A.; Neitzert M.; Oberdörfer Y.; Fuchs H. Force Spectroscopy of Molecular Systems—Single Molecule Spectroscopy of Polymers and Biomolecules. Angew. Chem.-Int. Ed. 2000, 39 (18), 3212–3237. 10.1002/1521-3773(20000915)39:18<3212::AID-ANIE3212>3.0.CO;2-X. 11028062

[ref120] Dudko O. K.; Hummer G.; Szabo A. Theory, Analysis, and Interpretation of Single-Molecule Force Spectroscopy Experiments. Proc. Natl. Acad. Sci. U. S. A. 2008, 105 (41), 15755–15760. 10.1073/pnas.0806085105. 18852468 PMC2572921

[ref121] Li N.; Zhang L.; Qiao O.; Wang X.; Xu L.; Gong Y. Special Contribution of Atomic Force Microscopy in Cell Death Research. Nanotechnol. Rev. 2024, 13 (1), 20230208 10.1515/ntrev-2023-0208.

[ref122] Razvag Y.; Gutkin V.; Reches M. Probing the Interaction of Individual Amino Acids with Inorganic Surfaces Using Atomic Force Spectroscopy. Langmuir 2013, 29 (32), 10102–10109. 10.1021/la4015866. 23859476

[ref123] Zou S.; Schönherr H.; Vancso G. J. Force Spectroscopy of Quadruple H-Bonded Dimers by AFM: Dynamic Bond Rupture and Molecular Time- Temperature Superposition. J. Am. Chem. Soc. 2005, 127 (32), 11230–11231. 10.1021/ja0531475. 16089437

[ref124] Sulchek T. A.; Friddle R. W.; Langry K.; Lau E. Y.; Albrecht H.; Ratto T. V.; DeNardo S. J.; Colvin M. E.; Noy A. Dynamic Force Spectroscopy of Parallel Individual Mucin1-Antibody Bonds. Proc. Natl. Acad. Sci. U. S. A. 2005, 102 (46), 16638–16643. 10.1073/pnas.0505208102. 16269547 PMC1276867

[ref125] Puchner E. M.; Gaub H. E. Force and Function: Probing Proteins with AFM-Based Force Spectroscopy. Curr. Opin. Struct. Biol. 2009, 19 (5), 605–614. 10.1016/j.sbi.2009.09.005. 19822417

[ref126] Smith B. L.; Schäffer T. E.; Viani M.; Thompson J. B.; Frederick N. A.; Kindt J.; Belcher A.; Stucky G. D.; Morse D. E.; Hansma P. K. Molecular Mechanistic Origin of the Toughness of Natural Adhesives, Fibres and Composites. Nature 1999, 399 (6738), 761–763. 10.1038/21607.

[ref127] Li H.; Linke W. A.; Oberhauser A. F.; Carrion-Vazquez M.; Kerkvliet J. G.; Lu H.; Marszalek P. E.; Fernandez J. M. Reverse Engineering of the Giant Muscle Protein Titin. Nature 2002, 418 (6901), 998–1002. 10.1038/nature00938. 12198551

[ref128] Higgins M. J.; Molino P.; Mulvaney P.; Wetherbee R. The Structure and Nanomechanical Properties of the Adhesive Mucilage That Mediates Diatom-Substratum Adhesion and Motility. J. Phycol. 2003, 39 (6), 1181–1193. 10.1111/j.0022-3646.2003.03-027.x.

[ref129] Fantner G. E.; Oroudjev E.; Schitter G.; Golde L. S.; Thurner P.; Finch M. M.; Turner P.; Gutsmann T.; Morse D. E.; Hansma H.; Hansma P. K. Sacrificial Bonds and Hidden Length: Unraveling Molecular Mesostructures in Tough Materials. Biophys. J. 2006, 90 (4), 1411–1418. 10.1529/biophysj.105.069344. 16326907 PMC1367291

[ref130] Giannotti M. I.; Vancso G. J. Interrogation of Single Synthetic Polymer Chains and Polysaccharides by AFM-Based Force Spectroscopy. ChemPhysChem 2007, 8 (16), 2290–2307. 10.1002/cphc.200700175. 17847140

[ref131] Wei H.; van de Ven T. G. AFM-Based Single Molecule Force Spectroscopy of Polymer Chains: Theoretical Models and Applications. Appl. Spectrosc. Rev. 2008, 43 (2), 111–133. 10.1080/05704920701831254.

[ref132] Hughes M. L.; Dougan L. The Physics of Pulling Polyproteins: a Review of Single Molecule Force Spectroscopy Using the AFM to Study Protein Unfolding. Rep. Prog. Phys. 2016, 79 (7), 076601 10.1088/0034-4885/79/7/076601. 27309041

[ref133] Dugdale T.; Dagastine R.; Chiovitti A.; Mulvaney P.; Wetherbee R. Single Adhesive Nanofibers from a Live Diatom Have the Signature Fingerprint of Modular Proteins. Biophys. J. 2005, 89 (6), 4252–4260. 10.1529/biophysj.105.062489. 16169972 PMC1366990

[ref134] Lee H.; Scherer N. F.; Messersmith P. B. Single-Molecule Mechanics of Mussel Adhesion. Proc. Natl. Acad. Sci. U. S. A. 2006, 103 (35), 12999–13003. 10.1073/pnas.0605552103. 16920796 PMC1559742

[ref135] Li Y.; Qin M.; Li Y.; Cao Y.; Wang W. Single Molecule Evidence for the Adaptive Binding of DOPA to Different Wet Surfaces. Langmuir 2014, 30 (15), 4358–4366. 10.1021/la501189n. 24716607

[ref136] Zhang J.; Lei H.; Qin M.; Wang W.; Cao Y. Quantifying Cation-Π Interactions in Marine Adhesive Proteins Using Single-Molecule Force Spectroscopy. Supramolecular Materials 2022, 1, 100005 10.1016/j.supmat.2021.100005.

[ref137] Xu L.-C.; Siedlecki C. A. Effects of Surface Wettability and Contact Time on Protein Adhesion to Biomaterial Surfaces. Biomaterials 2007, 28 (22), 3273–3283. 10.1016/j.biomaterials.2007.03.032. 17466368 PMC3671914

[ref138] Sui X.; Zapotoczny S.; Benetti E. M.; Schön P.; Vancso G. J. Characterization and Molecular Engineering of Surface-Grafted Polymer Brushes across the Length Scales by Atomic Force Microscopy. J. Mater. Chem. 2010, 20 (24), 4981–4993. 10.1039/b924392e.

[ref139] Schön P.; Kutnyanszky E.; ten Donkelaar B.; Santonicola M. G.; Tecim T.; Aldred N.; Clare A. S.; Vancso G. J. Probing Biofouling Resistant Polymer Brush Surfaces by Atomic Force Microscopy Based Force Spectroscopy. Colloid Surf. B-Biointerfaces 2013, 102, 923–930. 10.1016/j.colsurfb.2012.09.021. 23138001

[ref140] Wu T.; Efimenko K.; Genzer J. Preparing High-Density Polymer Brushes by Mechanically Assisted Polymer Assembly. Macromolecules 2001, 34 (4), 684–686. 10.1021/ma001750w.

[ref141] Wang J.; Wei J. Hydrogel Brushes Grafted from Stainless Steel Via Surface-Initiated Atom Transfer Radical Polymerization for Marine Antifouling. Appl. Surf. Sci. 2016, 382, 202–216. 10.1016/j.apsusc.2016.03.223.

[ref142] Chen Y.; Liu D.; Deng Q.; He X.; Wang X. Atom Transfer Radical Polymerization Directly from Poly (Vinylidene Fluoride): Surface and Antifouling Properties. J. Polym. Sci., Part A: Polym. Chem. 2006, 44 (11), 3434–3443. 10.1002/pola.21456.

[ref143] Guo S.; Jańczewski D.; Zhu X.; Quintana R.; He T.; Neoh K. G. Surface Charge Control for Zwitterionic Polymer Brushes: Tailoring Surface Properties to Antifouling Applications. J. Colloid Interface Sci. 2015, 452, 43–53. 10.1016/j.jcis.2015.04.013. 25913777

[ref144] Kaholek M.; Lee W.-K.; Ahn S.-J.; Ma H.; Caster K. C.; LaMattina B.; Zauscher S. Stimulus-Responsive Poly (N-Isopropylacrylamide) Brushes and Nanopatterns Prepared by Surface-Initiated Polymerization. Chem. Mater. 2004, 16 (19), 3688–3696. 10.1021/cm049562y.

[ref145] Kaholek M.; Lee W.-K.; Feng J.; LaMattina B.; Dyer D. J.; Zauscher S. Weak Polyelectrolyte Brush Arrays Fabricated by Combining Electron-Beam Lithography with Surface-Initiated Photopolymerization. Chem. Mater. 2006, 18 (16), 3660–3664. 10.1021/cm060276r.

[ref146] Kobayashi M.; Terayama Y.; Kikuchi M.; Takahara A. Chain Dimensions and Surface Characterization of Superhydrophilic Polymer Brushes with Zwitterion Side Groups. Soft Matter 2013, 9 (21), 5138–5148. 10.1039/c3sm27700c.

[ref147] Zhao C.; Li L.; Wang Q.; Yu Q.; Zheng J. Effect of Film Thickness on the Antifouling Performance of Poly (Hydroxy-Functional Methacrylates) Grafted Surfaces. Langmuir 2011, 27 (8), 4906–4913. 10.1021/la200061h. 21405141

[ref148] Lau K. H. A.; Sileika T. S.; Park S. H.; Sousa A. M.; Burch P.; Szleifer I.; Messersmith P. B. Molecular Design of Antifouling Polymer Brushes Using Sequence-Specific Peptoids. Adv. Mater. Interfaces 2015, 2 (1), 1400225 10.1002/admi.201400225. 26167449 PMC4497591

[ref149] Volkov D.; Strack G.; Halámek J.; Katz E.; Sokolov I. Atomic Force Microscopy Study of Immunosensor Surface to Scale Down the Size of Elisa-Type Sensors. Nanotechnology 2010, 21 (14), 145503 10.1088/0957-4484/21/14/145503. 20234083

[ref150] Sui X.; Chen Q.; Hempenius M. A.; Vancso G. J. Probing the Collapse Dynamics of Poly (N-Isopropylacrylamide) Brushes by AFM: Effects of Co-Nonsolvency and Grafting Densities. Small 2011, 7 (10), 1440–1447. 10.1002/smll.201002229. 21506265

[ref151] Dokukin M. E.; Kuroki H.; Minko S.; Sokolov I. AFM Study of Polymer Brush Grafted to Deformable Surfaces: Quantitative Properties of the Brush and Substrate Mechanics. Macromolecules 2017, 50 (1), 275–282. 10.1021/acs.macromol.6b02149.

[ref152] Teunissen L. W.; Kuzmyn A. R.; Ruggeri F. S.; Smulders M. M.; Zuilhof H. Thermoresponsive, Pyrrolidone-Based Antifouling Polymer Brushes. Adv. Mater. Interfaces 2022, 9 (6), 2101717 10.1002/admi.202101717.

[ref153] Parnell A. J.; Martin S. J.; Jones R. A.; Vasilev C.; Crook C. J.; Ryan A. J. Direct Visualization of the Real Time Swelling and Collapse of a Poly (Methacrylic Acid) Brush Using Atomic Force Microscopy. Soft Matter 2009, 5 (2), 296–299. 10.1039/B812872C.

[ref154] Willott J. D.; Murdoch T. J.; Webber G. B.; Wanless E. J. Nature of the Specific Anion Response of a Hydrophobic Weak Polyelectrolyte Brush Revealed by AFM Force Measurements. Macromolecules 2016, 49 (6), 2327–2338. 10.1021/acs.macromol.5b02656.

[ref155] LeMieux M. C.; Lin Y. H.; Cuong P. D.; Ahn H. S.; Zubarev E. R.; Tsukruk V. V. Microtribological and Nanomechanical Properties of Switchable Y-Shaped Amphiphilic Polymer Brushes. Adv. Funct. Mater. 2005, 15 (9), 1529–1540. 10.1002/adfm.200500088.

[ref156] Martinelli E.; Agostini S.; Galli G.; Chiellini E.; Glisenti A.; Pettitt M. E.; Callow M. E.; Callow J. A.; Graf K.; Bartels F. W. Nanostructured Films of Amphiphilic Fluorinated Block Copolymers for Fouling Release Application. Langmuir 2008, 24 (22), 13138–13147. 10.1021/la801991k. 18928304

[ref157] Kim K. S.; Gunari N.; MacNeil D.; Finlay J.; Callow M.; Callow J.; Walker G. C. Aqueous-Based Fabrication of Low-Voc Nanostructured Block Copolymer Films as Potential Marine Antifouling Coatings. ACS Appl. Mater. Interfaces 2016, 8 (31), 20342–20351. 10.1021/acsami.6b04629. 27388921

[ref158] Xie Q. Y.; Zeng H. H.; Peng Q. M.; Bressy C.; Ma C. F.; Zhang G. Z. Self-Stratifying Silicone Coating with Nonleaching Antifoulant for Marine Anti-Biofouling. Adv. Mater. Interfaces 2019, 6 (13), 1900535 10.1002/admi.201900535.

[ref159] Jiang S.; Cao Z. Ultralow-Fouling, Functionalizable, and Hydrolyzable Zwitterionic Materials and Their Derivatives for Biological Applications. Adv. Mater. 2010, 22 (9), 920–932. 10.1002/adma.200901407. 20217815

[ref160] Banerjee S. L.; Bhattacharya K.; Samanta S.; Singha N. K. Self-Healable Antifouling Zwitterionic Hydrogel Based on Synergistic Phototriggered Dynamic Disulfide Metathesis Reaction and Ionic Interaction. ACS Appl. Mater. Interfaces 2018, 10 (32), 27391–27406. 10.1021/acsami.8b10446. 30084628

[ref161] Yang W.; Pan M.; Zhang J.; Zhang L.; Lin F.; Liu X.; Huang C.; Chen X.-Z.; Wang J.; Yan B.; Zeng H. A Universal Strategy for Constructing Robust and Antifouling Cellulose Nanocrystal Coating. Adv. Funct. Mater. 2022, 32 (8), 2109989 10.1002/adfm.202109989.

[ref162] Lu D.; Wang H.; Li T.; Li Y.; Dou F.; Sun S.; Guo H.; Liao S.; Yang Z.; Wei Q.; Lei Z. Mussel-Inspired Thermoresponsive Polypeptide-Pluronic Copolymers for Versatile Surgical Adhesives and Hemostasis. ACS Appl. Mater. Interfaces 2017, 9 (20), 16756–16766. 10.1021/acsami.6b16575. 28472883

[ref163] Yang Y.; Liang Y.; Chen J.; Duan X.; Guo B. Mussel-Inspired Adhesive Antioxidant Antibacterial Hemostatic Composite Hydrogel Wound Dressing Via Photo-Polymerization for Infected Skin Wound Healing. Bioact. Mater. 2022, 8, 341–354. 10.1016/j.bioactmat.2021.06.014. 34541405 PMC8427086

[ref164] Wang T.; Bai J.; Lu M.; Huang C.; Geng D.; Chen G.; Wang L.; Qi J.; Cui W.; Deng L. Engineering Immunomodulatory and Osteoinductive Implant Surfaces Via Mussel Adhesion-Mediated Ion Coordination and Molecular Clicking. Nat. Commun. 2022, 13 (1), 1–17. 10.1038/s41467-021-27816-1. 35013289 PMC8748715

[ref165] Xie C.; Wang X.; He H.; Ding Y.; Lu X. Mussel-Inspired Hydrogels for Self-Adhesive Bioelectronics. Adv. Funct. Mater. 2020, 30 (25), 1909954 10.1002/adfm.201909954.

[ref166] Yu Q.; Zheng Z.; Dong X.; Cao R.; Zhang S.; Wu X.; Zhang X. Mussel-Inspired Hydrogels as Tough, Self-Adhesive and Conductive Bioelectronics: a Review. Soft Matter 2021, 17 (39), 8786–8804. 10.1039/D1SM00997D. 34596200

[ref167] Geng H.; Zhong Q.-Z.; Li J.; Lin Z.; Cui J.; Caruso F.; Hao J. Metal Ion-Directed Functional Metal-Phenolic Materials. Chem. Rev. 2022, 122 (13), 11432–11473. 10.1021/acs.chemrev.1c01042. 35537069

[ref168] Duan W.; Bian X.; Bu Y. Applications of Bioadhesives: a Mini Review. Front. Bioeng. Biotechnol. 2021, 9, 716035 10.3389/fbioe.2021.716035. 34540814 PMC8446440

[ref169] Keating J.; McQueen M. Substitutes for Autologous Bone Graft in Orthopaedic Trauma. J. Bone Joint Surg.-Br. Vol. 2001, 83B (1), 3–8. 10.1302/0301-620X.83B1.0830003. 11245534

[ref170] Han L.; Lu X.; Wang M.; Gan D.; Deng W.; Wang K.; Fang L.; Liu K.; Chan C. W.; Tang Y.; et al. A Mussel-Inspired Conductive, Self-Adhesive, and Self-Healable Tough Hydrogel as Cell Stimulators and Implantable Bioelectronics. Small 2017, 13 (2), 1601916 10.1002/smll.201601916. 27779812

[ref171] Masud N.; Rade J.; Hasib M. H. H.; Krishnamurthy A.; Sarkar A. Machine Learning Approaches for Improving Atomic Force Microscopy Instrumentation and Data Analytics. Front. Physics 2024, 12, 1347648 10.3389/fphy.2024.1347648.

[ref172] Krull A.; Hirsch P.; Rother C.; Schiffrin A.; Krull C. Artificial-Intelligence-Driven Scanning Probe Microscopy. Communications Physics 2020, 3 (1), 54 10.1038/s42005-020-0317-3.

[ref173] Kocur V.; Hegrová V.; Patočka M.; Neuman J.; Herout A. Correction of AFM Data Artifacts Using a Convolutional Neural Network Trained with Synthetically Generated Data. Ultramicroscopy 2023, 246, 113666 10.1016/j.ultramic.2022.113666. 36599269

[ref174] Müller P.; Abuhattum S.; Möllmert S.; Ulbricht E.; Taubenberger A. V.; Guck J. Nanite: Using Machine Learning to Assess the Quality of Atomic Force Microscopy-Enabled Nano-Indentation Data. BMC Bioinformatics 2019, 20 (1), 465 10.1186/s12859-019-3010-3. 31500563 PMC6734308

[ref175] Zhou F.; Wang W.; Li M.; Liu L. Force Curve Classification Using Independent Component Analysis and Support Vector Machine. 2015 9th IEEE International Conference on Nano/Molecular Medicine & Engineering (NANOMED); IEEE: 2015; pp 167–172.

[ref176] Ilieva N. I.; Galvanetto N.; Allegra M.; Brucale M.; Laio A. Automatic Classification of Single-Molecule Force Spectroscopy Traces from Heterogeneous Samples. Bioinformatics 2020, 36 (20), 5014–5020. 10.1093/bioinformatics/btaa626. 32653898

[ref177] Sokolov I.; Dokukin M.; Kalaparthi V.; Miljkovic M.; Wang A.; Seigne J.; Grivas P.; Demidenko E. Noninvasive Diagnostic Imaging Using Machine-Learning Analysis of Nanoresolution Images of Cell Surfaces: Detection of Bladder Cancer. Proc. Natl. Acad. Sci. U. S. A. 2018, 115 (51), 12920–12925. 10.1073/pnas.1816459115. 30509988 PMC6304950

[ref178] Xu X.; Feng H.; Zhao Y.; Shi Y.; Feng W.; Loh X. J.; Vancso G. J.; Guo S. AFM-Based Nanomechanics and Machine Learning for Rapid and Non-Destructive Detection of Bacterial Viability. Cell Rep. Phys. Sci. 2024, 5 (4), 101902 10.1016/j.xcrp.2024.101902.

[ref179] Karuth A.; Szwiec S.; Casanola-Martin G. M.; Khanam A.; Safaripour M.; Boucher D.; Xia W.; Webster D. C.; Rasulev B. Integrated Machine Learning, Computational, and Experimental Investigation of Compatibility in Oil-Modified Silicone Elastomer Coatings. Prog. Org. Coat. 2024, 193, 108526 10.1016/j.porgcoat.2024.108526.

